# Independent origins and evolution of the secondary replicons of the class Gammaproteobacteria

**DOI:** 10.1099/mgen.0.001025

**Published:** 2023-05-15

**Authors:** Christopher Riccardi, Piotr Koper, Gabriel Innocenti, George C. diCenzo, Marco Fondi, Alessio Mengoni, Elena Perrin

**Affiliations:** ^1^​ Department of Biology, University of Florence, Via Madonna del Piano 6, 50019, Sesto Fiorentino (FI), Italy; ^2^​ Department of Genetics and Microbiology, Maria Curie-Skłodowska University, Akademicka 19, 20-033, Lublin, Poland; ^3^​ Center for Cancer Research, Medical University of Vienna, Vienna, Austria; ^4^​ Department of Biology, Queen’s University, 116 Barrie Street, Kingston, Ontario, K7L 3N6, Canada

**Keywords:** Gammaproteobacteria, multipartite genomes, secondary replicons, *Vibrionaceae*, *Pseudoalteromonas*, chromid

## Abstract

Multipartite genomes, consisting of more than one replicon, have been found in approximately 10 % of bacteria, many of which belong to the phylum Proteobacteria. Many aspects of their origin and evolution, and the possible advantages related to this type of genome structure, remain to be elucidated. Here, we performed a systematic analysis of the presence and distribution of multipartite genomes in the class Gammaproteobacteria, which includes several genera with diverse lifestyles. Within this class, multipartite genomes are mainly found in the order Alteromonadales (mostly in the genus *Pseudoalteromonas *) and in the family *Vibrionaceae *. Our data suggest that the emergence of secondary replicons in Gammaproteobacteria is rare and that they derive from plasmids. Despite their multiple origins, we highlighted the presence of evolutionary trends such as the inverse proportionality of the genome to chromosome size ratio, which appears to be a general feature of bacteria with multipartite genomes irrespective of taxonomic group. We also highlighted some functional trends. The core gene set of the secondary replicons is extremely small, probably limited to essential genes or genes that favour their maintenance in the genome, while the other genes are less conserved. This hypothesis agrees with the idea that the primary advantage of secondary replicons could be to facilitate gene acquisition through horizontal gene transfer, resulting in replicons enriched in genes associated with adaptation to different ecological niches. Indeed, secondary replicons are enriched both in genes that could promote adaptation to harsh environments, such as those involved in antibiotic, biocide and metal resistance, and in functional categories related to the exploitation of environmental resources (e.g. carbohydrates), which can complement chromosomal functions.

## Data Summary

All custom scripts and main datasets are available in three open repositories at https://github.com/combogenomics/scripts_for_the_people/tree/master/multigenomeLoader, https://github.com/koper86/Gamma_proteo_multipartite and https://github.com/chrisondakeys/Gamma_proteo_multipartite.

The authors confirm all supporting data, code and protocols have been provided within the article or through supplementary data files.

**Impact Statement**

Multipartite genomes in bacteria are rare and many questions about the benefits they can confer to the harbouring cells remain unanswered. Our data highlight evolutionary trends in secondary replicons despite their multiple origins, confirming a possible role in the colonization of new environments. In particular, we suggest that the metabolic advantages related to the presence of secondary replicons could explain their appearance in some bacterial groups.

## Introduction

Bacteria with multipartite genomes possess at least one additional large replicon of 350 kb or larger, in addition to the main chromosome [1, 2]. Approximately 10 % of the currently fully sequenced and assembled bacterial genomes are multipartite. Secondary replicons are classified as chromids or secondary chromosomes when they encode essential genes, while they are defined as megaplasmids when they are dispensable, hence lacking essential genes. While several *in silico* metrics have been proposed as proxies for classification of a secondary replicon as a chromid, proper classification requires experimental validation of its essentiality. Each replicon in a genome usually differs in the functional classification of its genes, its evolutionary trajectory, and its rates of evolution and mutation, with secondary replicons generally displaying higher variability at both the gene and the nucleotide levels (see [1] for a review of multipartite genomes). Several hypotheses have been put forth to describe the possible advantages of multipartite genomes [1]. For example, we recently suggested that the primary advantage of secondary replicons is that they increase rates of gene gain through horizontal gene transfer, consequently resulting in replicons enriched in genes associated with adaptation to new environments [2]. Additionally, Sonnenberg *et al*. [3] proposed that the presence of two large replicons in *Vibrionaceae* allows spatial separation of different gene categories inside the cell, and that there is a link between the position of a gene and its function.

Bacteria with multipartite genomes have been found in phylogenetically distant species, but their presence is particularly abundant in the phylum Proteobacteria [1, 4]. In particular, this genome organization has been found in several genera within the class Alphaproteobacteria (e.g. *Azospirillum *, *Agrobacterium *, *Brucella *, *Mesorhizobium *, *Methylobacterium *, *Novasphingobium*, *Rhizobium* and *Sinorhizobium *) [1], as well as the genera *Burkholderia *, *Paraburkholderia *, *Cupriavidus* and *Ralstonia* of the class Betaproteobacteria [2]. In the class Gammaproteobacteria, multipartite genomes have mainly been identified in the family *Vibrionaceae* and in the genus *Pseudoalteromonas* [1, 4] and, in both cases, the secondary replicons were proposed to have originated from plasmids [5, 6].

To date, all the sequenced genomes belonging to the family *Vibrionaceae* are multipartite, with the exception of some *Vibrio cholerae* strains in which a fusion between the main chromosome and the secondary replicon is likely to have occurred [7]. In addition, *V. cholerae* has been used as a model system to study the mechanisms of replication of secondary replicons (see the introduction of [8] for a nice review of this topic). Members of the family *Vibrionaceae* are characterized by a variety of life styles [9]. Indeed, this family is widely distributed in aquatic environments (freshwater, estuarine and marine ecosystems, as well as aquaculture systems), where they contribute to nutrient cycling [10], but they can also live associated with a host as pathogens (several species are pathogens for fish, shellfish, coral and mammals) [11] or as bioluminescent symbionts of marine fishes and squids [9, 12]. Some lesser-known species are psychrophiles, piezophiles, or halophiles [13].

Members of the genus *Pseudoalteromonas* are marine bacteria specialized in surface-associated habitats, but they can also be found in deep sea, polar waters, sea ice and temperate salterns. They are biofilm formers and are found both on ocean particles and on the surfaces of marine eukaryotes [14–16]. In the genus *Pseudoalteromonas *, most of the sequenced genomes have a secondary replicon, and it has recently been demonstrated that these replicons are replicated unidirectionally, with the exception of the chromids of *P. spongiae* and *P. piratica *, which are replicated bidirectionally [17]. Through a phylogenetic and a timescale analysis, Liao *et al*. [18] showed that a secondary replicon and the chromosome were probably present in the common ancestor of *Pseudoalteromonas* and evolved together, and some evidence suggests that this secondary replicon originated from a megaplasmid [6, 17–20].

An interesting observation is that in both *Vibrionaceae* and *Pseudoalteromonas* the pangenome categories show spatial organization, both in terms of their distribution along a replicon and between replicons [3, 6]. Indeed, on the chromosome, there is an overrepresentation of core and softcore genes around the *ori*, and overrepresentation of shell and cloud genes around the *ter* region [3, 6]. All gene categories are much more evenly distributed across secondary replicons in the family *Vibrionaceae* [3], while in *Pseudoalteromonas* core/softcore genes are significantly overrepresented in late replicating sectors of the secondary replicon, regardless of how it is replicated [6]. These differences could be related to the specialized roles of secondary replicons, which have been shaped by the acquired and retained set of genes [6].

In this work, we investigated the distribution of multipartite genomes with the class Gammaproteobacteria. In total, more than 2000 genomes were analysed and the presence of secondary replicons was assessed for each of them. By studying the phylogenetic signal of specific marker sequences, we hypothesized the origin of each secondary replicon previously identified in several genera. Finally, the roles of genes harboured by these additional replicons was studied to unveil their contribution to the functional repertoire of the cell and to assess whether specific gene categories tend to be overrepresented on these extra-chromosomal DNA molecules.

## Methods

### Classification of DNA replicons

The complete genomes of 2323 Gammaproteobacteria were downloaded from the Refseq database (October 2020), and the replicons classified based on sequence properties as done previously [1] using a custom pipeline described in [2] and available at https://github.com/combogenomics/scripts_for_the_people/tree/master/multigenomeLoader. Organisms with more than one DNA molecule were annotated as ‘multipartite’. Additional information on metadata is given in Table S1, available in the online version of this article.

### Phylogenetic analysis of the Gammaproteobacteria

A maximum-likelihood phylogeny of representative Gammaproteobacteria was constructed as follows. Alignment seeds for 30 highly conserved protein markers (Supplementary_Materials) [21] were downloaded from the Pfam database [22] and converted to hidden Markov models (HMMs) using HMMER version 3.1b2 [23]. HMMER was then used to search each HMM against all downloaded proteomes. Subsequently, the top hits in each proteome were searched against the entire Pfam protein FASTA database (last accessed 15 November 2021) [22] using DIAMOND version 0.8.22 [24], and only those proteins whose top hit was to the protein class represented by the starting HMM were classified as true marker proteins. The 137 strains in which 1 or more marker protein was not identified were excluded from downstream analysis. The remaining 2190 strains were further filtered to limit the dataset to 1 random representative strain per species, and to remove *Candidatus* taxa and strains not assigned to a genus. In addition, several strains were removed during manual editing of the multiple sequence alignment (see below) due to them having a gappy and dishomogeneous signal throughout most of the alignment. This resulted in a final dataset of 1128 organisms.

A maximum-likelihood phylogeny was then built from the concatenated alignments of 25 marker proteins (Frr, Pgk, RplA, RplB, RplC, RplD, RplE, RplF, RplK, RplN, RplP, RplS, RplT, RpmA, RpoB, RpsB, RpsC, RpsE, RpsI, RpsJ, RpsK, RpsM, RpsS, SmpB, Tsf); 5 (DnaG, PyrG, RplM, NusA and RplL) of the initial 30 markers were excluded either due to large variation in sequence length or due to poor alignment quality (see Supplementary_Materials). Alignments of individual marker protein sets were performed using Mafft [25] version 7.205 in automatic mode, followed by manual inspection and trimming with the aid of BioEdit version 7.2.5 [26]. Following concatenation of the 25 alignments, RAxML version 8.2.9 [27] was used to build a maximum-likelihood phylogeny, using the LG amino acid substitution model and the GAMMA rate heterogeneity. The final tree is the bootstrap best tree following 200 bootstrap replicates, which was visualized using iTol [28]. To ensure that the limited number (25) of genes or that not considering recombination events did not produce a low-quality phylogeny, we compared our tree to that of the Genome Taxonomy Database (release R05-RS95, 17 July 2020) [29, 30]. The only major discrepancy was the position of the genus *Sodalis *, which was closer to the genus *Pectobacterium* in the Genome Taxonomy Database phylogeny. As this difference does not impact on the interpretation of our data, we proceeded with our phylogenetic analysis.

### Replicon phylogenetic signal retrieval

We collected the amino acid sequences and features information for 259 publicly available genome assemblies assigned to the order Alteromonadales and the family *Vibrionaceae *. A total of 25 genera were represented (19 belonging to the Alteromonodales and 6 belonging to the *Vibrionaceae *). A total of 145 secondary replicons were detected across the 259 assemblies, with 141 assemblies containing at least 1 secondary replicon. We also downloaded seed sequences for five protein family domains (Pfam) involved in replicon partitioning during cell division: ParA HTH_54 (PF18607), ParA AAA_31 (PF13614), ParBc (PF02195), Rep-3 (PF01051) and RPA (PF10134). Canonically, ParA homologues are ATPases and ParBc homologues are DNA-binding proteins [31].

HMMER was used to convert the markers’ multiple sequence alignment into a series of HMMs. The hmmsearch function of HMMER was used to search the proteome of each replicon for potential matches to each HMM. The top hit for each marker was kept for a subsequent, more thorough search against the entire Pfam database (last accessed 15 November 2021). This first step produced 404 positive matches for marker ParA AAA_31 (100 % of replicons), 211 for ParA HTH_54 (52.2 %), 398 for ParBc (98.5 %), 30 for Rep_3 (7.4 %) and 11 (2.7 %) for RPA. Given the low hit rates for Rep-3, ParA HTH_54 and RPA, we discarded these markers. The ParBc HMM returned hits for all replicons except NC_018679.1 (* Alteromonas macleodii* str. Balearic Sea AD45), NZ_AP019651.1 (* Vibrio taketomensis* C4III291), NZ_CP012738.1 (* Pseudoalteromonas* sp. 1_2015MBL_MicDiv), NZ_CP013021.1 (* Agarivorans gilvus* WH0801), NZ_CP013139.1 (* Pseudoalteromonas* sp. Bsw20308), NZ_CP041661.1 (* Catenovulum sediminis* WS1-A); we therefore excluded these six replicons from further analysis.

We next used DIAMOND with sensitive mode to search the proteins identified in the first step, against the entire Pfam database. Proteins whose top hit did not correspond to the protein family used to initially identify the protein were excluded from further analysis. Each set of orthologues were aligned using Mafft version 7.205 and alignments were inspected manually and trimmed using BioEdit. Maximum-likelihood phylogenetic trees were then computed using RAxML with the LG amino acid substitution model and the CAT rate heterogeneity. Additional details are available in the Supplementary_Materials.

### Accessory genes identification in the genus *Pseudoalteromonas* and the family *Vibrionaceae*

The CARD (for antibiotic genes) [32] and BacMet (for antibacterial biocide, and metal resistance genes) [33] protein databases were downloaded on 9 June 2022 and converted to a DIAMOND database [34]. The DIAMOND searches were performed separately for the chromosomes and secondary replicons of each taxonomic group of interest, using default options. In order to work with a non-redundant dataset, proteins were clustered using a 95 % identity threshold using Cd-hit version 4.6 [35] with no additional options, and only one representative sequence per cluster was kept for further analysis. Chi-squared tests were performed using R on the reduced dataset.

### Pangenome calculation

All genomes were annotated using Prokka v. 1.13 [36] with default parameters to ensure consistent gene calling and annotation across analysed data. Pangenomes were then calculated using Roary v. 3.11 [37]. Separate pangenomes were calculated for the chromosomes and for each category of extrachromosomal replicon in each genus. To determine the optimal value of the minimum percentage identity for blastp for each of the pangenomes, calculations were made using identity thresholds in the range of 40–90 %, and then the relationship between this minimum percentage identity and the number of core and accessory genes was plotted. For all analysed genera, the pangenomes obtained with a 60 % identity threshold were used, since, as shown in Fig. S1, this proved to be the most appropriate threshold, providing a reasonable trade-off between correctly identifying true orthologues and limiting the number of non-orthologous genes being incorrectly grouped as orthologues.

### Functional reannotation and analyses

Representative amino acid sequences were obtained for all genes found in each genus–replicon pangenome using in-house scripts and reannotated using eggNOG-mapper v. 2.1.15 [38] with the -d flag set to 'bact'. The accessory pangenome was defined as the genes found in <95 % of the strains. Data for the respective genes were extracted from the eggNOG database (COG categories, KO number assignment).

### Statistical tests and plotting

The statistical significance of differences in the percentage share of individual functional categories between replicons within a genus was verified using Fisher’s exact test. All functional data were handled and visualized using pandas, matplotlib and seaborn python libraries. Default parameters were used for all software unless specified otherwise.

### Scripts availability

All custom scripts are available in open repositories at https://github.com/combogenomics/scripts_for_the_people/tree/master/multigenomeLoader, https://github.com/koper86/Gamma_proteo_multipartite and https://github.com/chrisondakeys/Gamma_proteo_multipartite.

## Results

### Multipartite genomes in the Gammaproteobacteria are mainly found in the order Alteromonadales and in the family *Vibrionaceae*

**Fig. 1.**
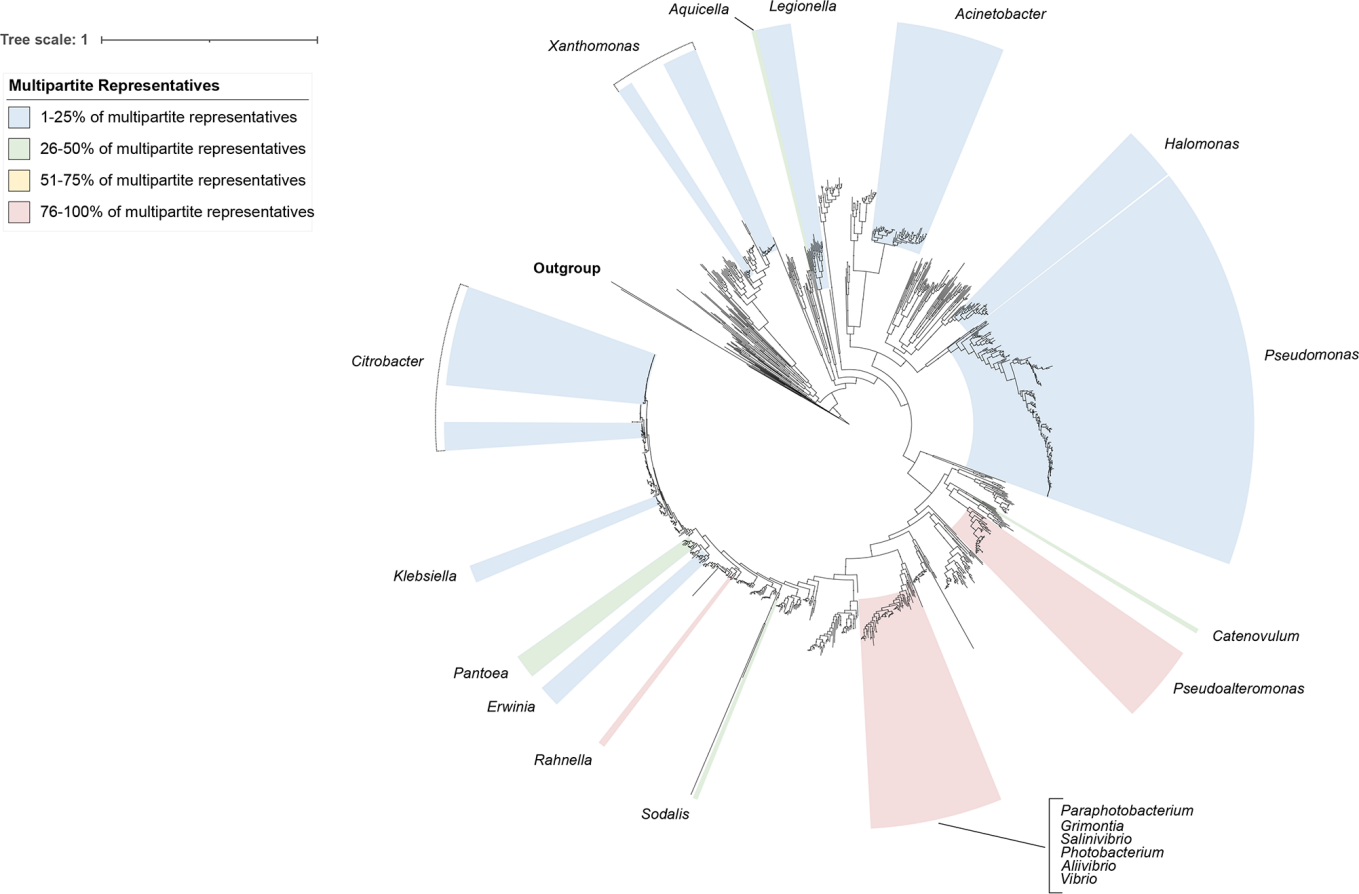
Distribution of multipartite genomes in Gammaproteobacteria. Maximum-likelihood phylogeny of 5256 distinct alignment patterns for 1128 non-redundant species. Labels and colours refer to genera where at least 1 % of the publicly available genome sequences are multipartite. Multipartite genomes are found across several orders of the class Gammaproteobacteria, but the majority of organisms with multipartite genomes fall within the order Alteromonadales and family *Vibrionaceae *. Bootstrap support values of at least 90 % are reported in Fig. S2.

When we started our analysis (December 2020), 2323 complete and closed genomes belonging to the class Gammaproteobacteria were available through the National Center for Biotechnology Information (NCBI) genome database (Table S1), representing 244 genera. Twenty-two of these genera included at least one strain with a multipartite genome, meaning they carried at least two replicons of 350 kb or larger, with the largest secondary replicon having a length of ~2.8 Mb (Figs 1, S2 and Table S1). In some of these genera (* Acinetobacter *, *Citrobacter *, *Erwinia *, *Halomonas *, *Klebsiella *, *Legionella *, *Pseudomonas *, *Salmonella *, *Xanthomonas *), only a few strains (<25 %) had a representative with a multipartite genome (highlighted in blue in Figs 1, S2 and Table S1), while between 26 and 50 % of the strains in the genera *Aquicella *, *Sodalis* and *Pantoea* had multipartite genomes (highlighted in green in Figs 1 and S2). Furthermore, the genome of *Candidatus* Thiodictyon (not reported in the phylogenetic tree as explained in the Supplementary_Materials) and the four sequenced genomes belonging to the genus *Rahnella* are all multipartite (highlighted in red in Figs 1 and S2).

Overall, the vast majority of multipartite genomes identified in the Gammaproteobacteria were found in the order Alteromonadales and in the family *Vibrionaceae* (Table S1). In the Alteromonadales, the multipartite genomes were limited to the genus *Pseudoalteromonas *, where 35 out of the 40 (87.5 %) genomes were multipartite (highlighted in red in Figs 1 and S2), with an additional single multipartite genome in a representative of the genus *Catenovulum *. By contrast, all members of genera belonging to the family *Vibrionaceae* (* Aliivibrio *, *Grimontia *, *Paraphotobacterium *, *Photobacterium *, *Salinivibrio* and *Vibrio *) carried multipartite genomes (highlighted in red in Figs 1 and S2).

Although we did not attempt to classify secondary replicons as chromids or megaplasmids, a previous study suggested that, with a few rare exceptions, chromids within the Gammaproteobacteria are limited to the family *Vibrionaceae* and the genera *Pseudoaltermonas* and *Rahnella* [1]. When considered together with our observations, these data suggest that whereas the presence of megaplasmids shows species-to-species variation, chromids tend to be maintained across all members of a genus or higher taxonomic group, consistent with other studies [2, 4].

### Chromosome size is smaller in multipartite genomes in Gammaproteobacteria

**Fig. 2.**
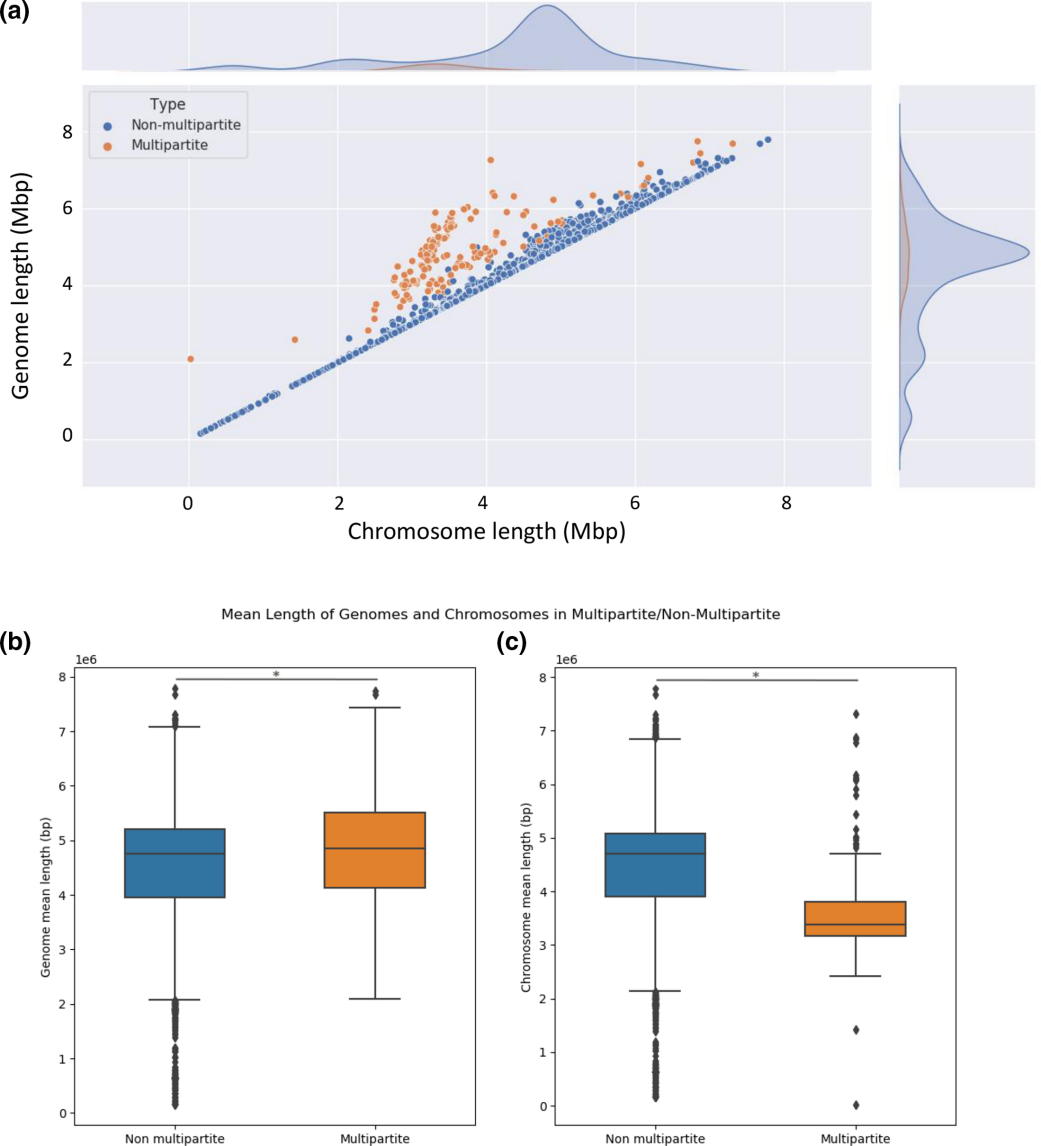
Genome and chromosome size in multipartite and non-multipartite genomes. (a) Scatter plot showing the distribution of all 2323 gammaproteobacterial genomes (non-multipartite genomes also include genomes that have plasmids <350 kb in size). (b, c) Boxplots showing (b) the average genome length in all multipartite and non-multipartite genomes (F-test, *P*<0.001) and (c) the mean chromosome length (F-test, *P*<0.001).

**Fig. 3.**
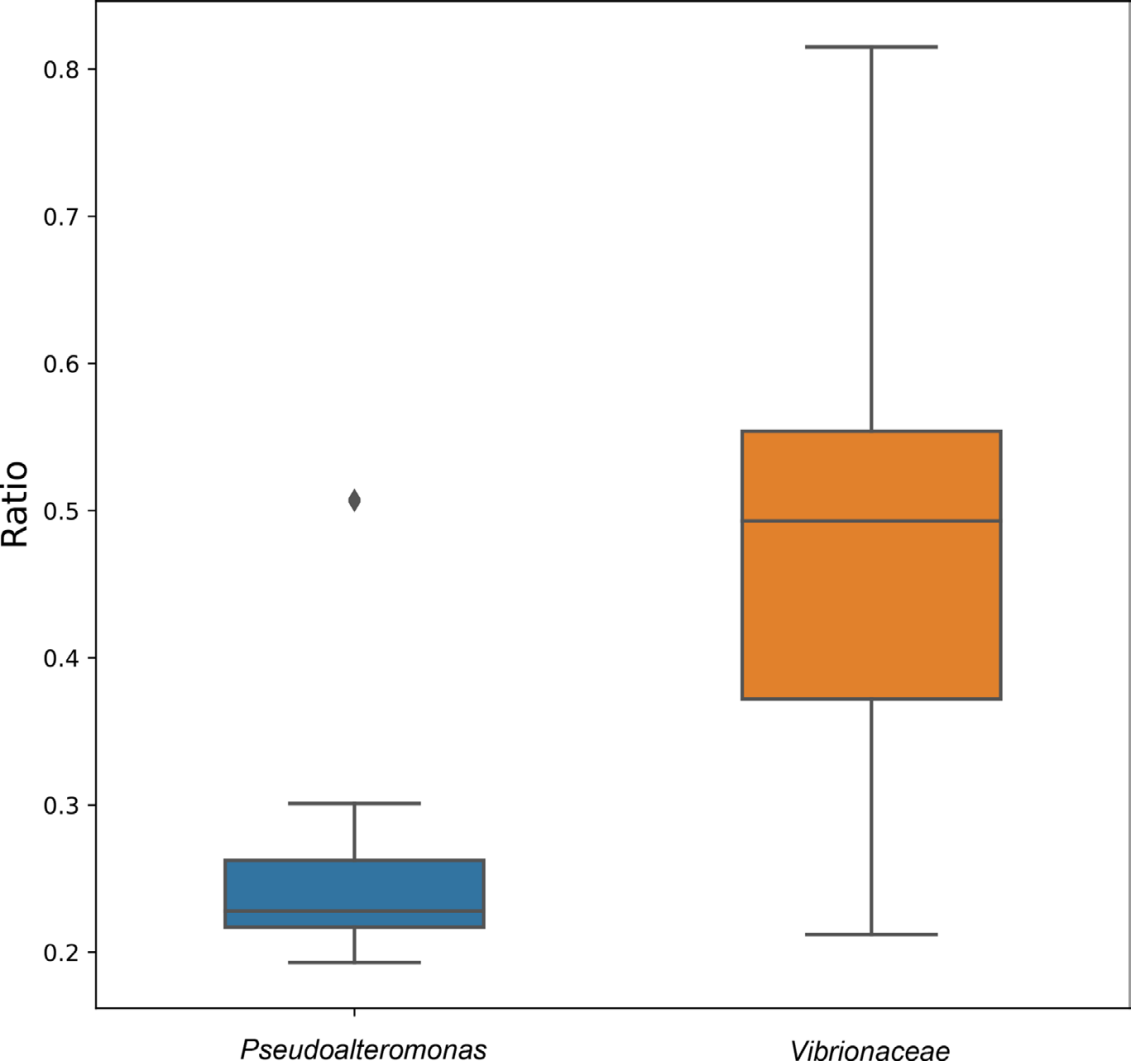
Ratio between the sum of secondary replicon sizes and chromosome sizes. Boxplot showing the average ratio of the sum of the sizes of all secondary replicons in a genome to that of the primary chromosome in the same assembly. The plot contains data from 140 observations: 35 *Pseudoalteromonas* and 105 *Vibrionaceae *.

The genome size of the Gammaproteobacteria included in our dataset varies between 157 543 bp (*Candidatus Carsonella ruddii* HT isolate Thao2000) and 7 783 862 bp (* Granulosicoccus antarcticus* IMCC3135) (Fig. 2a, Table S1). As previously reported for other taxonomic groups, Gammaproteobacteria with a multipartite genome have an average genome size that is slightly larger than that of Gammaproteobacteria without a multipartite genome (4.9 versus 4.4 Mb, median genome length 4.8 versus 4.7 Mb) (Fig. 2b) [1, 4]. Moreover, we observed that the average chromosome size of Gammaproteobacteria with multipartite genomes is significantly smaller than that of Gammaproteobacteria without multipartite genome (3.6 versus 4.4 Mb; F-test, *P*<0.001) (Fig. 2c), as was also reported for the Betaproteobacteria [2]. In the genus *Pseudoalteromonas* and in the family *Vibrionaceae *, the average ratio between the sizes of secondary replicons and chromosomes was 0.26 and 0.47, respectively (median ratio 0.23 and 0.49, respectively) (Figs 3 and S3). We also observed that the ratio of replicon sizes was relatively consistent across the genus *Pseudoalteromonas *, while the ratio was highly variable in the family *Vibrionaceae *, mainly due to variation in the size of the secondary replicons.

### Multipartite genomes in the order Alteromonadales and the family *Vibrionaceae* originated from independent events

**Fig. 4.**
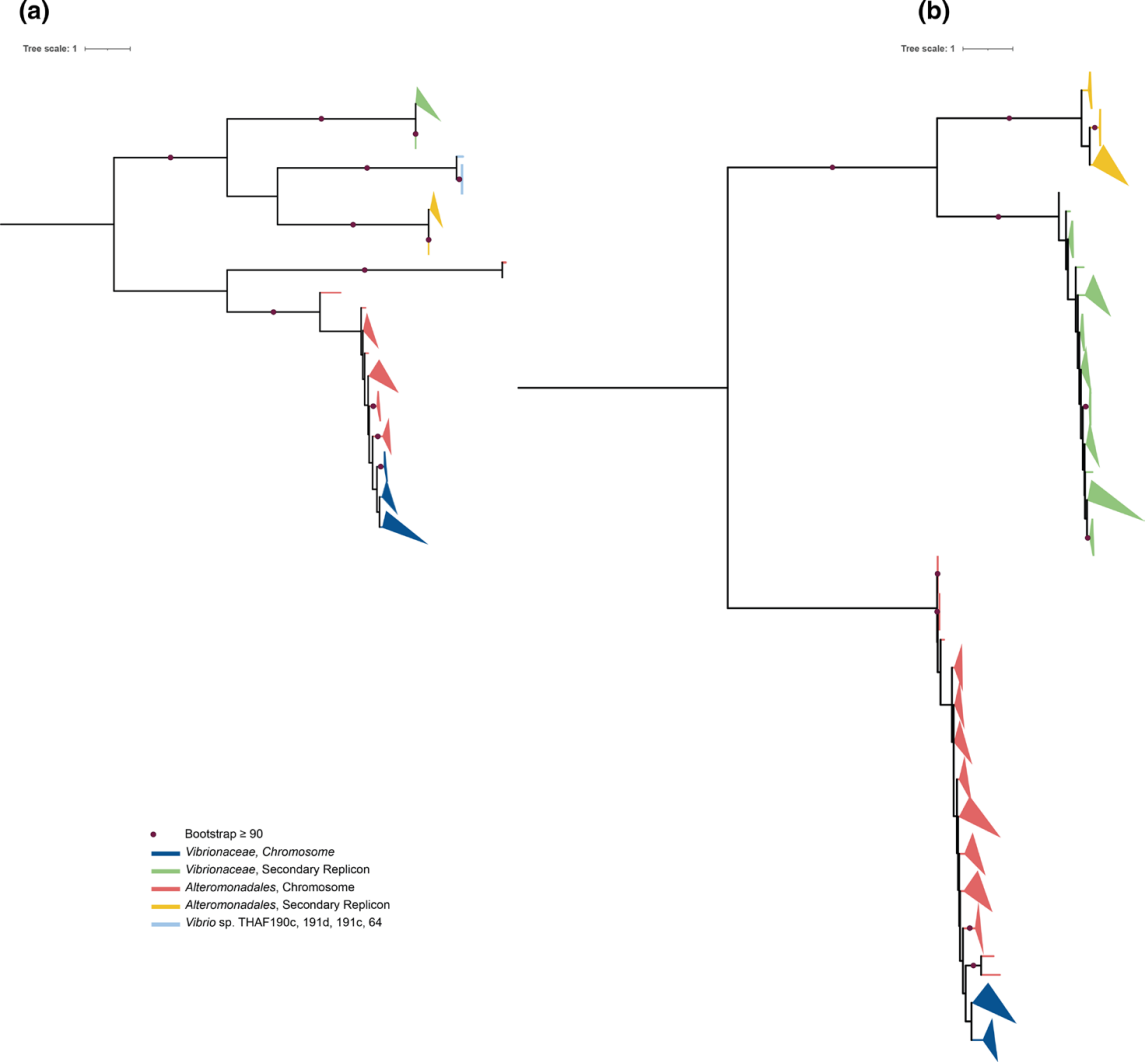
Phylogenetic relationship of the replicons of the order Alteromonodales and the family *Vibrionaceae.* Maximum-likelihood phylogeny based on (a) 236 sites of the partitioning protein ParA from 397 replicons or (b) 202 sites of the partitioning protein ParB from 387 replicons. Clades whose average branch length distance to their relative leaves is <0.7 are collapsed.

Since most of the multipartite genomes identified in the Gammaproteobacteria were within the order Alteromonadales and the family *Vibrionaceae *, subsequent analyses were focused on these two taxonomic groups; we excluded the genus *Rahnella* from downstream analyses due to the limited sample size. Phylogenetic trees of all replicons (chromosomes and secondary replicons) of the order Alteromonadales and the family *Vibrionaceae* were reconstructed on the basis of the partitioning proteins ParA (Fig. 4a) and ParB (Fig. 4b). In both phylogenies, the large secondary replicons were clearly separated from the chromosomes, suggesting that the secondary replicons did not evolve from chromosomes within these taxa, but more likely evolved from plasmids [1]. More notably, all of the secondary replicons (likely chromids) of the order Alteromonadales formed a monophyletic group, and likewise all secondary replicons (likely chromids) of the family *Vibrionaceae* formed their own monophyletic groups, with the exception of the second secondary replicons (likely megaplasmids) of *Vibrio* sp. THAF190c, THAF191c, THAF191d and THAF64 (Fig. 4 and Figs S4–8). These data suggest that all putative chromids of the order Alteromonadales and the family *Vibrionaceae* arose from just two evolutionary events: one in the ancestor of the genus *Pseudoalteromonas* (as previously reported by [6]) and one in the common ancestor of the family *Vibrionaceae* (consistent with previous work [39]).

### The core gene set of secondary replicons is extremely small

The core gene set (defined as genes found in at least 95 % of the strains) of all the secondary replicons of the family *Vibrionaceae* (excluding the 4 additional replicons of *Vibrio* _sp._THAF190c, THAF191c, THAF191d and THAF64) consists of only 22 genes, ranging from 0.9–4.2 % of the total number of genes present on these replicons (Table S2). These genes include *parAB* encoding a replicon partitioning system, as well as transcriptional regulators and metabolic genes such as the essential genes *ddl* (d-alanine-d-alanine ligase, involved in d-amino acid metabolism and peptidoglycan biosynthesis) and *pdxH* (pyridoxamine 5′-phosphate oxidase, involved in vitamin B6 metabolism and biosynthesis of cofactors) [40–43]. The 4 additional replicons of *Vibrio* _sp._THAF190c, THAF191c, THAF191d and THAF64 share 32 genes, ranging from 6.5–8.6 % of the total number of genes present in these replicons (Table S3), including a type II toxin–antitoxin system that may contribute to the maintenance of these replicons.

In the genus *Pseudoalteromonas* the secondary replicons share 66 genes, ranging from 4.5–12.4 % of the total number of genes of these replicons (Table S4), which is consistent with past work [6]. These genes include *parAB*, *minCDE*, whose gene products regulate cell division, regulatory genes and a gene coding for a DNA-binding protein (*hupB*), as well as several metabolic genes coding for histidine biosynthesis (*hisFAHBCDG*), purine and pyrimidine metabolisms (although most have a homologue in the main chromosome), an acetolactate synthase (*ilvBH*) and a biopolymer transport system (*tonB–exbB–exbD*).

### Secondary replicons share a common functional gene set and genus-specific functional enrichments

**Fig. 5.**
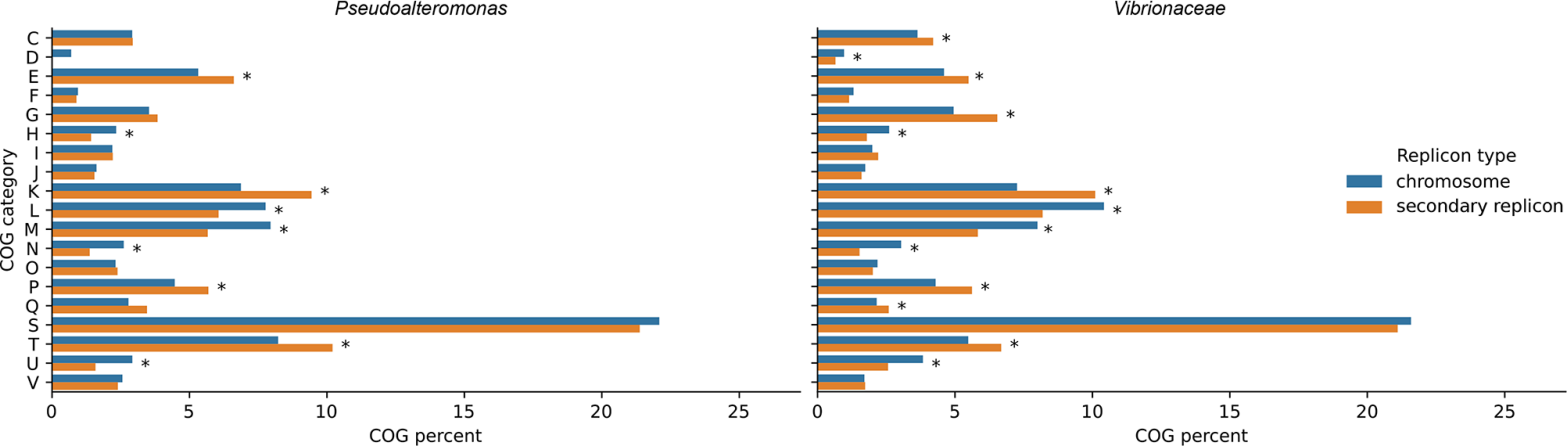
Bar plots showing COG category abundances in replicon-specific accessory pangenomes. Each sub-plot concerns data from a different taxon (data for individual genera within the family *Vibrionaceae* are reported in Fig. S9). The individual bars correspond to the percentage of gene clusters assigned to a specific COG category (blue, accessory pangenome of the chromosomes; orange, accessory pangenome of the secondary replicons). Asterisks symbolize comparisons for which the difference was statistically significant (*P*<0.05 in Fisher’s exact test). COG categories: C (energy production and conversion), D (cell cycle control, cell division, chromosome partitioning), E (amino acid transport and metabolism), F (nucleotide transport and metabolism), G (carbohydrate transport and metabolism), H (coenzyme transport and metabolism), I (lipid transport and metabolism), J (translation, ribosomal structure and biogenesis), K (transcription), L (replication, recombination and repair), M (cell wall/membrane/envelope biogenesis), N (cell motility), O (posttranslational modification, protein turnover, chaperones), P (inorganic ion transport and metabolism), Q (secondary metabolite biosynthesis, transport and catabolism), S (function unknown), T (signal transduction mechanisms), U (intracellular trafficking, secretion, vesicular transport), V (defence mechanisms).

A replicon-specific pangenome functional annotation (with the accessory pangenome defined as the genes found in <95 % of the strains) revealed the presence of a shared set of functional categories differentially enriched in the chromosome and secondary replicons (Figs 5, S9 and Table S5). In particular, COG categories H (coenzyme metabolism), L (replication and repair), M (cell wall/membrane/envelop biogenesis), N (cell motility) and U (intracellular trafficking and secretion) are enriched in the chromosomal accessory pangenomes of both the family *Vibrionaceae* and the genus *Pseudoalteromonas* (Figs 5, S9 and Table S5), suggesting a reduced occurrence of central cellular and metabolic functions in the secondary replicons. In addition, COG category D (cell cycle control and mitosis) is enriched in the chromosomal accessory pangenome of the family *Vibrionaceae* (Figs 5, S9 and Table S5). In contrast, COG categories E (amino acid metabolism and transport), K (transcription), P (inorganic ion transport and metabolism) and T (signal transduction) represent a shared set of enriched functions in the secondary replicon accessory pangenomes of the family *Vibrionaceae* and the genus *Pseudoalteromonas *. This may suggest a role in nutrition and regulation in response to environmental (nutritional) stimuli of secondary replicons in these two taxonimic groups. The secondary replicons of the family *Vibrionaceae* are also enriched for COG categories C (energy production and conversion) and G (carbohydrate metabolism and transport), consistent with these replicons putatively being involved in metabolic adaptation to environmental variation.

A similar analysis was performed on the abundance of KEGG categories in the chromosome and the secondary replicon accessory pangenomes (Fig. S10, Table S6). A common functional enrichment for the KEGG category ‘metabolism of cofactors and vitamins’ was detected on the chromosomes of both the family *Vibrionaceae* and the genus *Pseudoalteromonas *. The categories ‘protein families: metabolism’, ‘protein families: genetic information processing’ and ‘unclassified: genetic information processing in the chromosome’ are also enriched in the chromosomes of the family *Vibrionaceae *. On the other hand, the category ‘carbohydrate metabolism’ is enriched on the secondary replicons of the family *Vibrionaceae *, while the category ‘protein families: signalling and cellular processes’ is enriched on the secondary replicons of the genus *Pseudoalteromonas *.

### Secondary replicons help adaptation of ***Pseudoalteromonas*** **and** ***Vibrionaceae*** **to harsh environments**

**Fig. 6.**
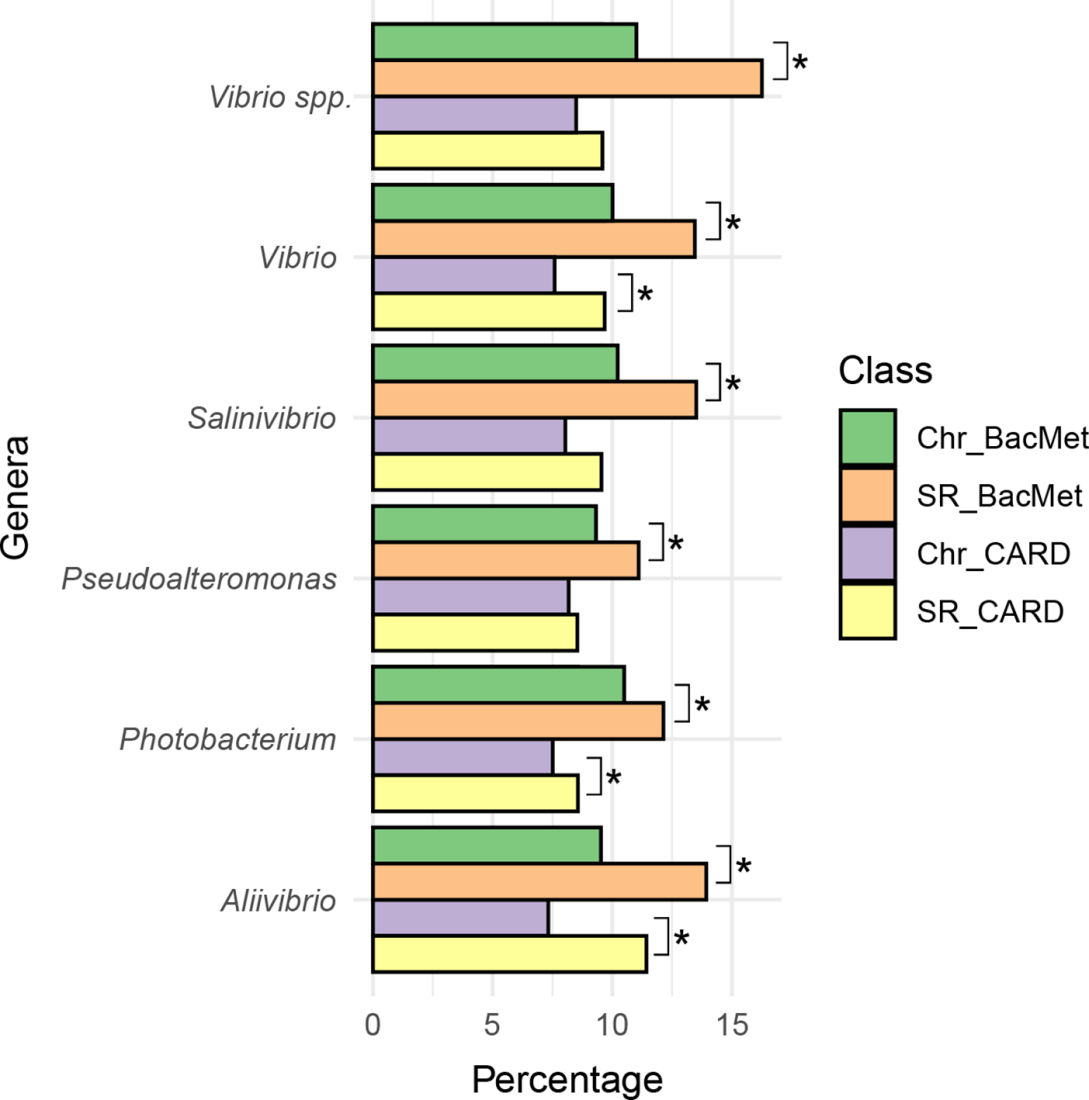
Bar plots showing CARD and BacMet gene abundances in each replicon. The chromosomes and secondary replicons of Alteromonadales and *Vibrionaceae* were queried for the presence of antibiotic resistance and metal resistance genes using the CARD and BacMet databases, respectively. The percentage reported in each sub-plot derives from the ratio between the number of positive matches from each database in the non-redundant body of genes in every genus. In relation to each database, and under the assumption that the presence of resistance genes is independent of the DNA molecule classification (chromosome or secondary replicon), the asterisks identify where the probability that a chi-square value this large, or larger, would occur by chance is <5 % (i.e. *P*<0.05). The sub-plot labelled ‘* Vibrio* spp.’ represents the comparison between the additional secondary replicons present in *Vibrio* spp. THAF190c, THAF191c, THAF191d and THAF64 and the relative chromosomes. Green, chromosome results with BacMet; violet, chromosome results with CARD; orange, secondary replicon results with BacMet; yellow, secondary replicon results with CARD.

Finally, since secondary replicons are known to carry ‘accessory genes’ that confer an indirect benefit to themselves by improving the fitness of the bacterium that hosts them [44], such as genes involved in virulence, antibiotic resistance or biofilm production, we evaluated the presence of genes involved in antibiotic, biocide and metal resistance in the chromosomes and the secondary replicons. We found that genes involved in biocide and heavy metal resistance are enriched in secondary replicons in both the genus *Pseudoalteromonas* and the family *Vibrionaceae *, while genes involved in antibiotic resistance are enriched in the secondary replicons of the genera *Aliivibrio *, *Photobacterium* and *Vibrio* (Fig. 6).

## Discussion

In contrast to the classical model of the bacterial genome consisting of a single replicon (the chromosome) and occasionally small, dispensable accessory replicons (plasmids), several bacteria display a multipartite genome structure, where the genome is composed of several large replicons. These replicons include the chromosome, large and dispensable plasmids known as megaplasmids, and chromids that carry essential genes and are thought to have evolved from megaplasmids [1, 4]. A survey of genomes deposited in public databases suggested that approximately 10 % of bacterial genomes are multipartite and that this genome organization mostly occurs within species that interact with eukaryotic hosts in classes Alphaproteobacteria, Betaproteobacteria and Gammaproteobacteria [1, 4]. In this work, we performed a comprehensive analysis of the presence and distribution of multipartite genomes in the Gammaproteobacteria, a class that includes several genera of bacteria that have commensal, symbiotic, or pathogenic interactions with a variety of eukaryotic hosts, including plants, animals and humans. Our analysis revealed the presence of at least 1 strain with a secondary replicon (350 kb size or larger [1, 2]) in 22 of the 244 genera included in our dataset. In most cases, only a few representatives of each genus possess a multipartite genome, with past work suggesting that these are likely megaplasmids; together, these data suggest multiple independent gains and losses of megaplasmids across individual genera of the class Gammaproteobacteria. On the other hand, secondary replicons were found in most members of the genus *Pseudoaltermonas* and all members of the family *Vibrionaceae* and the genus *Rahnella *, consistent with previous studies [6, 11]. This may be due to these taxa carrying chromids instead of (or in addition to) megaplasmids. Indeed, chromids are thought to be better conserved and may help to define new genera [1, 4]. Our data favour the hypothesis that the chromids of the genus *Pseudoaltermonas* and the family *Vibrionaceae* originated from just two independent events: one in the ancestor of the genus *Pseudoalteromonas* and one in the common ancestor of all the *Vibrionaceae *. The presence of secondary replicons in several species, but deriving from a few events, has also previously been reported for Alphaproteobacteria and Betaproteobacteria [2, 4]. Our data for Gammaproteobacteria support the hypothesis that the emergence of secondary replicons in Proteobacteria is rare, and that their abundance in the Proteobacteria is driven primarily by vertical transmission.

It has been suggested that the multipartite genome structure allows for an increase in bacterial genome size, while maintaining a relatively fast generation time due to DNA replication initiating from several independent replicons in parallel [45]. In both Alphaproteobacteria and Betaproteobacteria, a larger genome size has indeed been observed in strains with a multipartite genome, in comparison with those that do not have this type of structure [1, 2]. Moreover, a downward trend in chromosome size was also observed in bacteria with a multipartite structure compared to those without it [2, 46]. Our data are consistent with those studies and revealed an inverse relationship between genome size and chromosome size in the Gammaproteobacteria, suggesting that these trends may be general features of bacteria with multipartite genomes, irrespective of taxonomic groups. In particular, we found that chromosomes are significantly larger in Gammaproteobacteria without multipartite genomes than in strains with multipartite genomes. Moreover, we also observed that the ratio between the sum of the secondary replicon size and the chromosome size is relatively constant in the genus *Pseudoalteromonas *, while in the family *Vibrionaceae* the ratio is highly variable and driven by changes in the size of the secondary replicons. This higher variability in the family *Vibrionaceae* with respect to *Pseudoalteromonas* may reflect the high number of ecological niches [10] and lifestyles of *Vibrionaceae *, which affect the gene content of secondary replicons in terms of advantages/burdens [47, 48]. Alternatively, it may simply reflect the greater evolutionary distance between species in the family *Vibrionaceae* than in the genus *Pseudoaltermonas*.

We found that the core gene set of the secondary replicons is extremely small in both the genus *Pseudolateromonas* and the family *Vibrionaceae *. It includes genes involved in their replication and maintenance, like those encoding the ParAB partitioning system, for the control of cell division (*minCDE*), and regulatory and metabolic genes. The low number of shared genes suggests that there is high variation in gene content between strains, and that likely only genes essential for replicon maintenance or cell survival are highly conserved. This hypothesis is in agreement with our recent suggestion that the primary advantage of secondary replicons could be to facilitate gene acquisition through horizontal gene transfer, resulting in replicons enriched in genes associated with the adaptation to different ecological niches [2]. Indeed, the reduced number of essential genes in secondary replicons may make them more available for the integration of horizontally acquired DNA, since integration of new DNA has a lower probability of disrupting important genes [4, 49, 50]. Moreover, integration of genes into a secondary replicon may result in lower expression than if integrated into the chromosome, and low-expression horizontally acquired genes are more likely to be maintained than high-expression genes [2]. As an example, in the Alphaproteobacterium *Sinorhizobium meliloti *, the pSymB chromid was demonstrated to be essential for rhizosphere colonization, while the pSymA megaplasmid carries genes essential for nitrogen-fixing symbiosis with plants [51]. In the *Burkholderia cepacia* complex (of the class Betaproteobacteria) the third replicon, a megaplasmid, is associated with virulence, antifungal and proteolytic activity and plays a role in stress tolerance [52, 53]. In the family *Vibrionaceae* and the genus *Pseudoalteromonas *, we found that secondary replicons are enriched in genes involved in resistance to antibiotic, biocide and metal resistance, suggesting that these replicons could promote adaptation to harsh environments.

We also found that in both the family *Vibrionaceae* and the genus *Pseudoalteromonas* COG categories H, L, M and N are enriched in the chromosomal accessory pangenomes, while COG categories E, K, P and T are enriched in the accessory pangenomes of the secondary replicon. In addition, the COG categories C and G are enriched in the accessory pangenome of the secondary replicon of the family *Vibrionaceae *. COG categories E, K, P, T, C and G have also been reported to be enriched in the accessory pangenome of secondary replicons in other bacteria [1, 2]. Together, these observations support the hypothesis that secondary replicons may increase the functional complexity of the organism and provide functions associated with exploitation of environmental resources (e.g. carbohydrates), complementing chromosomal functions. Differential activation of gene expression and relevance of genes and metabolic pathways residing on secondary replicons for the exploitation of soil, plant rhizosphere and symbiosis have been shown for *S. meliloti *, where the two secondary replicons (pSymA and pSymB) are key for plant symbiosis and colonization of the rhizosphere [51, 54, 55]. It would be interesting to further explore this topic, for example by performing a comparative study of the simulated growth of genome-scale metabolic models of species with multipartite genomes in diverse environments, both as free-living organisms and during association with hosts. Such studies would allow researchers to test hypotheses related to the metabolic advantages of secondary replicons, while also considering the metabolic burden they may impose on the cell.

## Supplementary Data

Supplementary material 1

Supplementary material 2
