# Supplementary material for: Independent origins and evolution of the secondary replicons of the class Gammaproteobacteria
Source: Microb Genom. 2023 May 15;9(5):mgen001025. doi: 10.1099/mgen.0.001025 (PMC10272867; doi:10.1099/mgen.0.001025)
Supplement: Supplementary material 1 [file mgen-9-1025-s001.pdf]

Supplementary materials of "**Independent origins and evolution of the secondary replicons of the class Gammaproteobacteria**"

Multilocus sequence analysis (MLSA)

Data Collection

We intended to trace the evolutionary relationship that exists among species of the *γ-Proteobacteria*. We therefore considered 2323 members of this class, and four additional species to use as an outgroup during the construction of the tree. The outgroups were two *α-Proteobacteria*, namely *Rhodospirillum rubrum* and *Sinorhizobium meliloti*, and two members of the class Betaproteobacteria, *Chromobacterium violaceum* and *Ralstonia solanacearum*. For the task, the genomic nucleotide and amino acid sequences together with annotation files for 2327 organisms were downloaded from the NCBI using in-house scripts in Dec 2020. Alignment seeds for conserved markers were downloaded from the Pfam [1] database, and converted to Hidden Markov Profiles using HMMER version 3.1b2 [http://hmmer.org]. The entire Pfam FASTA database was downloaded through their FTP service on Nov 15 2021.

Dataset Filtering

We sought to identify phylogenetic signal through a Bidirectional Best Hits (BBH) approach, in which 30 conserved markers in the form of Hidden Markov Profiles are searched against the input proteomes, and the resulting matches are forwarded to a thorough search against the entire Pfam protein database. These markers consist of housekeeping genes that are involved in information processing (replication, transcription, and translation) or central metabolism, and thus are thought to be relatively recalcitrant to lateral gene transfer [2, 3].
These are listed below:

Gene	Description	Pfam
dnaG	DNA primase	PF01807,PF08275,PF08278,PF10410,PF13662
frr	ribosome recycling factor	PF01765
nusA	transcription elongation factor NusA	PF00575,PF08529,PF13184
pgk	phosphoglycerate kinase	PF00162
pyrG	CTP synthetase	PF00117,PF06418
rplA	50S ribosomal protein L1	PF00687
rplB	50S ribosomal protein L2	PF00181,PF03947
rplC	50S ribosomal protein L3	PF00297
rplD	50S ribosomal protein L4	PF00573
rplE	50S ribosomal protein L5	PF00281,PF00673
rplF	50S ribosomal protein L6	PF00347
rplK	50S ribosomal protein L11	PF00298,PF03946
rplL	50S ribosomal protein L7/L12	PF00542,PF16320
rplM	50S ribosomal protein L13	PF00572
rplN	50S ribosomal protein L14	PF00238
rplP	50S ribosomal protein L16	PF00252

Gene	Description	Pfam
rplS	50S ribosomal protein L19	PF01245
rplT	50S ribosomal protein L20	PF00453
rpmA	50S ribosomal protein L27	PF01016
rpoB	DNA-directed RNA polymerase subunit beta	PF00562,PF04560,PF04561,PF04563,PF04565,PF10385
rpsB	30S ribosomal protein S2	PF00318
rpsC	30S ribosomal protein S3	PF00189,PF07650
rpsE	30S ribosomal protein S5	PF00333,PF03719
rpsI	30S ribosomal protein S9	PF00380
rpsJ	30S ribosomal protein S10	PF00338
rpsK	30S ribosomal protein S11	PF00411
rpsM	30S ribosomal protein S13	PF00416
rpsS	30S ribosomal protein S19	PF00203
smpB	SsrA-binding protein	PF01668
tsf	elongation factor Ts	PF00889

Using this hybrid approach we were able to assign 30 markers in 94.1% of the entries (2190/2327). We then investigated case by case those species for which markers were not found, to see if at least one other representative of the genus was present in the data set. As a form of quality control, we filtered out possibly incomplete genomes that did not contain all of the marker proteins. The dataset was further filtered to include one random representative per species. Since hits for markers *dnaG* and *pyrG* were very heterogeneous, both in terms of the spread of sequence and domain composition, we decided not to take these two markers into account for the subsequent multiple sequence alignment stage. The final number of species included before the multiple alignments stage was 1139, with 28 eligible markers. While manually editing each MSA with BioEdit version 7.2.5 [4], we further excluded the following species, due to a gappy and dishomogeneous signal throughout most of the alignments:

Acinetobacter calcoaceticus isolate Acinetobacter calcoaceticus str. 2117, *Acinetobacter sp.* 10FS3-1, *Acinetobacter sp.* 323-1, *Klebsiella grimontii* NCTC9146 substr. serovar casular type 26 substr. serovar casular type 26, *Pantoea stewartii* ZJ-FGZX1, *Providencia alcalifaciens* NCTC10286, *Pseudomonas sp.* ADAK2, *Pseudomonas sp.* DG56-2, *Pseudomonas sp.* LG1E9, *Xenorhabdus doucetiae* FRM16, *Glaciecola sp.* THG-3.7.

And, for the same reason, the following markers:

rplM
nusA
rplL

From an initial number of 2327 species 1190 were removed, leaving a total of 1128 organisms of non redundant species and 25 markers, and 4392 distinct alignment patterns with a proportion of gaps of 0.0%. Multiple sequence alignments were performed using Mafft [5] version 7.205 in automatic mode; the RAxML version 8.2.9 algorithm [6] was used to build a maximum likelihood phylogeny, using the LG amino acid substitution model and the GAMMA rate heterogeneity. The final tree is the bootstrap best tree following 200 bootstrap replicates, which was visualized using iTol [7].

Replicon-level Phylogenetic Analyses

Data set description

We downloaded the amino acid sequences and features information for 259 publicly available species assigned to the orders of *Alteromonadales* and *Vibrionales*. A total of 25 genera were represented (19 and 6, from each order, respectively). 141/259 species had at least one secondary replicon assigned other than a primary

chromosome. The initial number of replicon amino acid sequences was 404. The largest and smallest replicon NZ_CP014782.1 and NZ_CP045357.1 counted 5192 and 364 sequences, respectively.

Signal Source Selection

We downloaded the Pfam seed sequences for five marker domains: ParA HTH_54 (PF18607), ParA AAA_31 (PF13614), ParBc (PF02195), Rep-3 (PF01051) and RPA (PF10134). These types of proteins play a role in localizing chromosome or plasmid DNA during the process of cell division, ensuring non-random distribution of DNA molecules into daughter cells. Canonically, ParA homologues are ATPases and ParBc homologues are DNA-binding proteins [8].

Phylogenetic Signal Retrieval

HMMER was used to convert the marker multiple sequence alignment into a series of Hidden Markov Profiles. The same software package was utilized using its default options to scan each replicon multi-FASTA with the profiles for a preliminary potential match list. The most significant hit for each marker was kept and the sequences thereof collected using in house scripts for a subsequent, more thorough search against the entire Pfam database (last accessed November 15th 2021). The preliminary step produced 404 positives for marker ParA AAA_31 (100% of replicons), 211 for ParA HTH_54 (52.2%), 398 for ParBc (98.5%), 30 for Rep_3 (7.4%) and 11 (2.7%) for RPA. HMMER search of marker ParBc did not return any match in replicons NC_018679.1 (*Alteromonas macleodii* str. 'Balearic Sea AD45'), NZ_AP019651.1 (*Vibrio taketomensis* C4III291), NZ_CP012738.1 (*Pseudoalteromonas* sp. 1_2015MBL_MicDiv), NZ_CP013021.1 (*Agarivorans gilvus* WH0801), NZ_CP013139.1 (*Pseudoalteromonas* sp. Bsw20308), NZ_CP041661.1 (*Catenovulum sed- iminis* WS1-A). Given the meager fraction of Rep_3, ParA HTH_54 and RPA matches we discarded these markers, excluded the six above-mentioned replicons from the analysis, and proceeded with the second it- eration of the BBH using DIAMOND version 0.8.22 enabling its mode designed for full sensitivity for hits of >40% identity [9]. The second search against the entire Pfam strongly confirmed what was already found with the first step, excluding only protein WP_086981293.1 (NZ_AP018690.1, *Vibrio aphrogenes* CA-1004) from the ParA AAA_31 domain matches. Three proteins were discarded for marker ParBc, namely WP_152470772.1 (NZ_CP045340.1, *Vibrio* sp. THAF190c), WP_152472879.1 (shared by three distinct *Vibrio* replicons NZ_CP045357.1, NZ_CP046067.1 and NZ_CP046164.1), and WP_165312997.1 (NZ_CP049332.1, *Vibrio* sp. ZWAL4003). A later investigation on these replicons indicated that they are the smallest replicons in the same assembly, and when we searched for the other secondary replicons we found a match for ParBc. Multiple sequence alignment was performed using Mafft.

ParA

The MSA was inspected manually using BioEdit. The following sequences were removed from the MSA since they carried only a partial portion of the phylogenetic signal, and presented erroneous substitutions in conserved sites:
WP_143873348.1 (NZ_CP041661.1, *Catenovulum* sediminis WS1-A)
WP_180826498.1 (NZ_CP030799.1, *Vibrio owensii* 20160513VC2W)
WP_155759773.1 (NZ_CP016352.1, *Vibrio natriegens* CCUG 16374)
WP_088135096.1 (NZ_CP018836.1, *Vibrio gazogenes* ATCC 43942)
the sequence WP_124745568.1 deriving from an anomalous assembly (NZ_CP034120.1, *Glaciecola amylolytica* THG-3.7)
and WP_089074682.1 (NZ_CP022356.1, *Paraphotobacterium marinum* NSCS20N07D)

After this filtering, the multiple sequence alignment was calculated again, and this time inspected and trimmed manually to hold as much of the informative signal as possible. The final number of alignment patterns was 236 on 397 replicons. The maximum likelihood phylogenetic tree was computed using RAxML with the LG amino acid substitution model and the CAT rate heterogeneity.

Secondary replicons of ParA

We isolated 139 ParA replicon-derived sequences from the initial data set and performed another multiple sequence alignment (same software and options, Mafft in automatic mode) using 348 distinct alignment patterns. As for the other markers, we manually inspected the MSA to exclude gappy regions and maximize the phylogenetic signal. We then fed the multiple sequence alignment to RAxML using the LG amino acid substitution model and the CAT rate heterogeneity.

ParBc

The same software was utilized for visualizing the multiple sequence alignment of ParBc. Some sequences were heavily displaced from the rest of the group in well conserved and clustered sites:
WP_024015777.1 (NC_023045.1, *Alteromonas mediterranea* MED64)
WP_180826314.1 (NZ_CP030799.1, *Vibrio owensii* 20160513VC2W)
WP_102524316.1 (NZ_LT960612.1, *Vibrio tapetis* subsp. tapetis CECT4600)
WP_128812496.1 (NZ_CP032094.1, *Vibrio alfacsensis* CAIM 1831) WP_089073750.1 (NZ_CP022355.1, *Paraphotobacterium marinum* NSCS20N07D) WP_124745567.1 (NZ_CP034120.1, *Glaciecola amylolytica* THG-3.7)

After removing the noisy sequences, we recomputed the alignment and performed manual trimming to keep as much of the conserved domain fraction as possible, for a final number of 387 replicon sequences and 202 sites. The maximum likelihood phylogenetic tree was computed using RAxML with the LG amino acid substitution model and the CAT rate heterogeneity.

Secondary replicons of ParBc

We isolated 134 ParBc replicon-derived sequences from the initial data set and performed another multiple sequence alignment (same software and options, Mafft in automatic mode) using 244 distinct alignment patterns. As for the other markers, we manually inspected the MSA to exclude gappy regions and maximize the phylogenetic signal. We then fed the multiple sequence alignment to RAxML using the LG amino acid substitution model and the CAT rate heterogeneity.

References

1. **Mistry J, Chuguransky S, Williams L, Qureshi M, Salazar GA, et al.** Pfam: The protein families database in 2021. Nucleic Acids Research 2021;49:D412–D419.
2. **Jain R, Rivera MC, Lake JA.** Horizontal gene transfer among genomes: The complexity hypothesis. Proceedings of the National Academy of Sciences 1999;96:3801–3806.
3. **Wu M, Scott AJ.** Phylogenomic analysis of bacterial and archaeal sequences with AMPHORA2. Bioinformatics 2012;28:1033–1034.
4. **Dagona AG.** BioEdit: a user-friendly biological sequence alignment editor and analysis program for Windows 95/98/NT. Nucleic acids symposium series. https://www.academia.edu/2034992/BioEdit_a_user_friendly_biological_sequence_alignment_editor_and_analysis_program_for_Windows_95_98_NT (1999, accessed 20 October 2022).
5. **Katoh K, Standley DM.** MAFFT Multiple Sequence Alignment Software Version 7: Improvements in Performance and Usability. Molecular Biology and Evolution 2013;30:772–780.
6. **Stamatakis A.** RAxML version 8: a tool for phylogenetic analysis and post-analysis of large phylogenies. Bioinformatics 2014;30:1312–1313.
7. **Letunic I, Bork P.** Interactive Tree Of Life (iTOL) v5: an online tool for phylogenetic tree display and annotation. Nucleic Acids Research 2021;49:W293–W296.
8. **Callaghan MM, Koch B, Hackett KT, Klimowicz AK, Schaub RE, et al.** Expression, Localization, and Protein Interactions of the Partitioning Proteins in the Gonococcal Type IV Secretion

System. Frontiers in Microbiology;12. https://www.frontiersin.org/article/10.3389/fmicb.2021.784483 (2021, accessed 13 May 2022).

9. **Buchfink B, Reuter K, Drost H-G.** Sensitive protein alignments at tree-of-life scale using DIAMOND. Nat Methods 2021;18:366–368.

Supplementary Figures Legend

Supplementary_Figure_1

The first subplot illustrates the relationship between the roary's selected identity threshold and the total number of orthologous gene clusters in the pangenomes of each genus. The second subgraph illustrates the number of core genes in relation to the selected identity threshold. The x-axis shows the identity threshold, and the y-axis is the total number of genes and the number of core genes, respectively.

Supplementary_Figure_2

Phylogeny of the *Gamma-Proteobacteria*.

Supplementary_Figure_3

Boxplot showing the average of the sum of sizes of all secondary replicons in a genome to that of the primary chromosome in the same assembly in the *Alteromonodales* order and in the *Vibrionaceae* family.

Supplementary_Figure_4

Phylogenetic relationship of the secondary replicons of the order *Alteromonodales* and *Vibrionales*. Maximum likelihood phylogeny on 348 sites for 139 secondary replicons using partitioning protein ParA AAA_31 (A) and 244 sites for 134 replicons using ParBc (B) as markers. Clades whose average branch length distance to their relative leaves is less than 0.7 were collapsed.

Supplementary_Figure_5

Expanded, unrooted phylogenetic tree on of all replicons for both orders. Bootstrap support greater or equal than 90 is reported; phylogenetic signal computed using partitioning protein ParA AAA31.

Supplementary_Figure_6

Expanded, unrooted phylogenetic tree of the secondary replicons for both orders. Bootstrap support greater or equal than 90 is reported; phylogenetic signal computed using partitioning protein ParA AAA31.

Supplementary_Figure_7

Expanded, unrooted phylogenetic tree of all replicons for both orders. Bootstrap support greater or equal than 90 is reported; phylogenetic signal computed using partitioning protein ParBc.

Supplementary_Figure_8

Expanded, unrooted phylogenetic tree of the secondary replicons for both orders. Bootstrap support greater or equal than 90 is reported; phylogenetic signal computed using partitioning protein ParBc.

Supplementary_Figure_9

Bar plots showing COG category abundances in replicon specific accessory pangenomes of each *Vibrionaceae* group. Each sub-plot concerns data from a different *Vibrionaceae* genus, while the sub-plot *Vibrio* spp. represents the comparison between the accessory pangenomes of chromosomes and the additional secondary replicons present in the *Vibrio* sp. THAF190c, THAF191c, THAF191d and THAF64 strains. The individual bars correspond to the percentage of gene clusters assigned to a specific COG category (blue - accessory chromosome pangenomes, orange - accessory secondary replicon pangenomes Asterisks symbolize comparisons for which the difference was statistically significant ($p < 0.05$ in Fischer's exact test).

Supplementary_Figure_10

Bar plots showing the abundance of KEGG categories in replicon specific accessory pangenomes.
Plots are similar to those shown in Figure 5 and Supplementary_Figure_9, but the annotation of specific gene clusters was performed based on the KEGG functional categories.

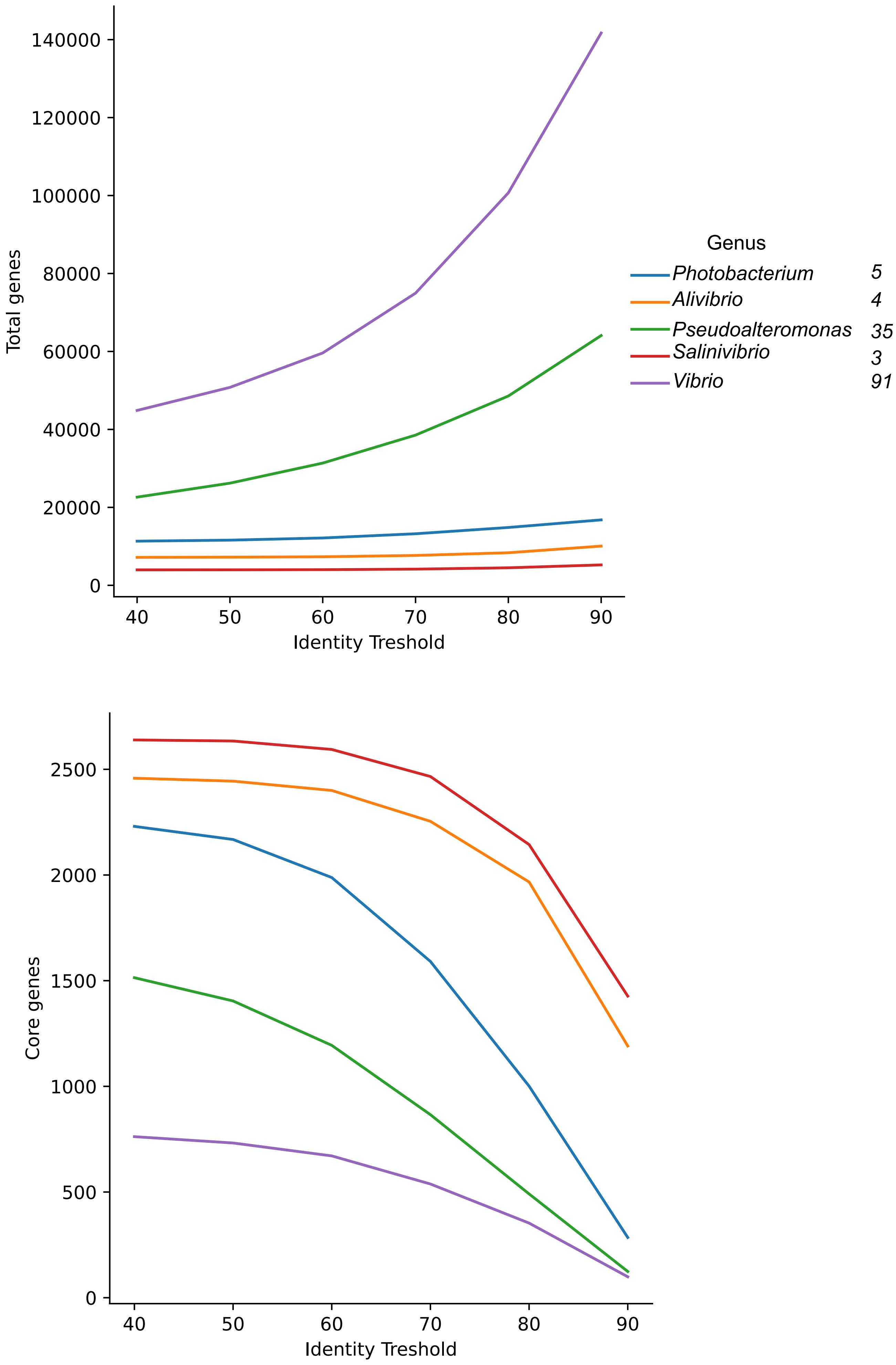

Supplementary_Figure_1

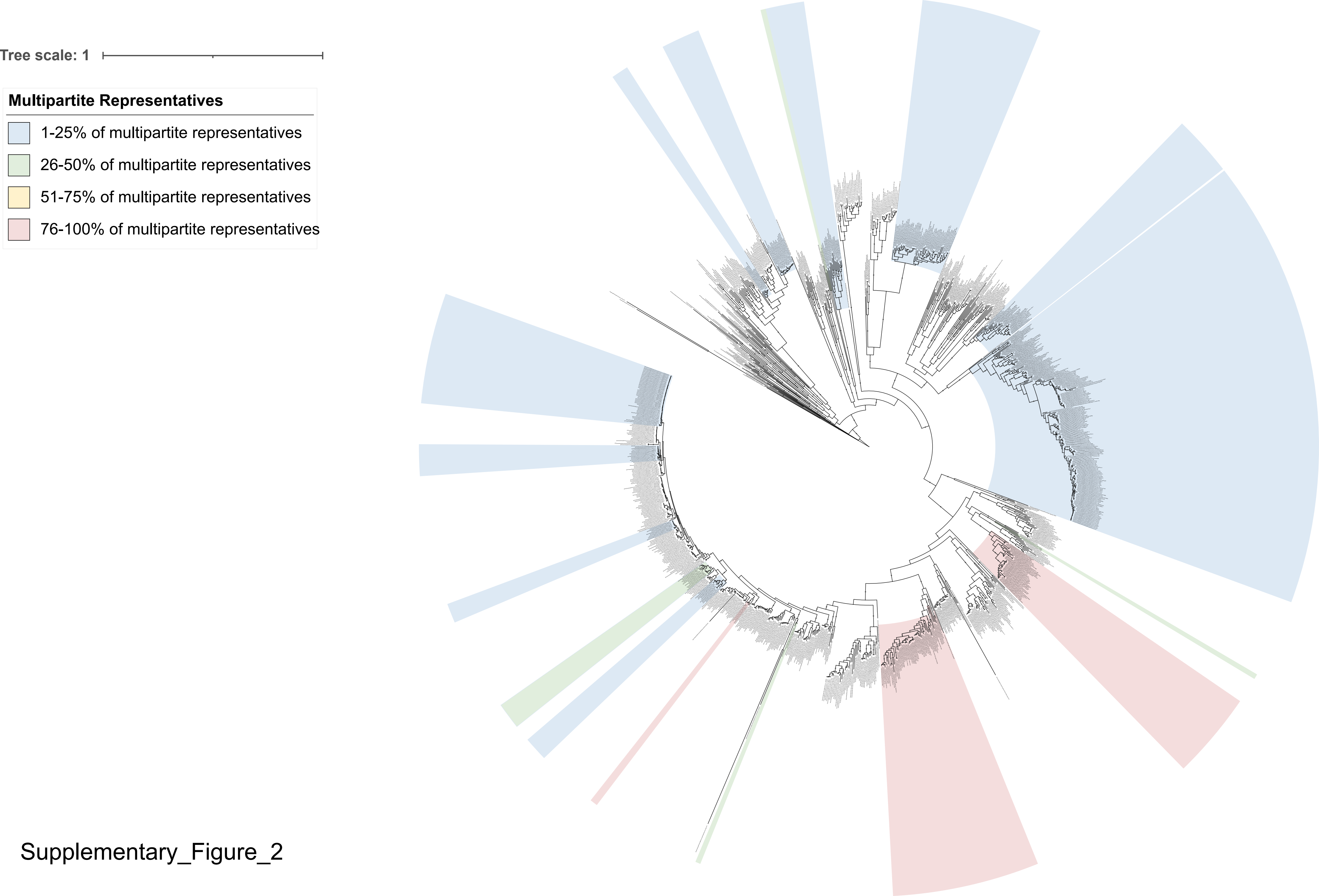

Supplementary_Figure_2

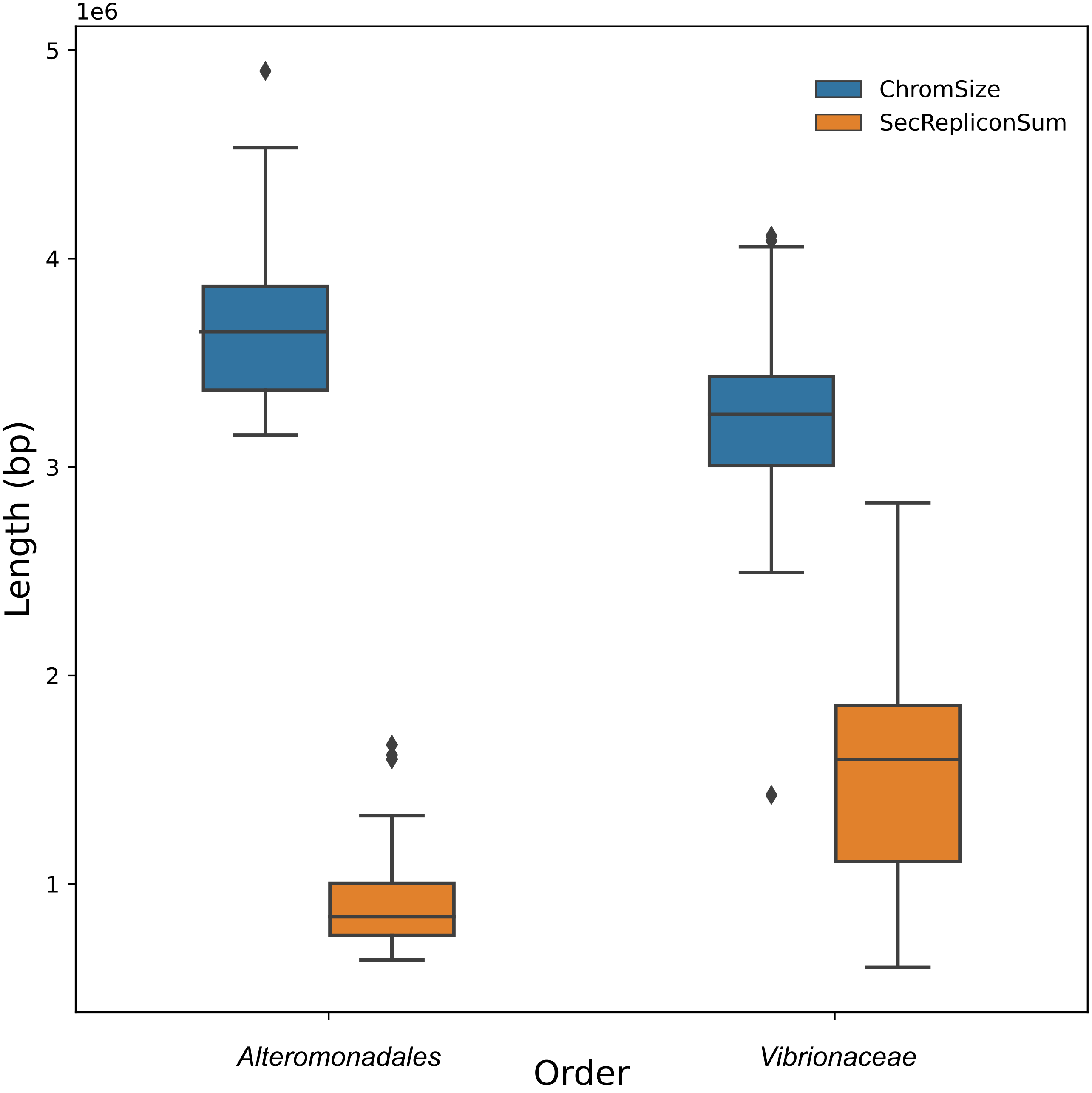

Supplementary_Figure_3

Supplementary_Figure_4

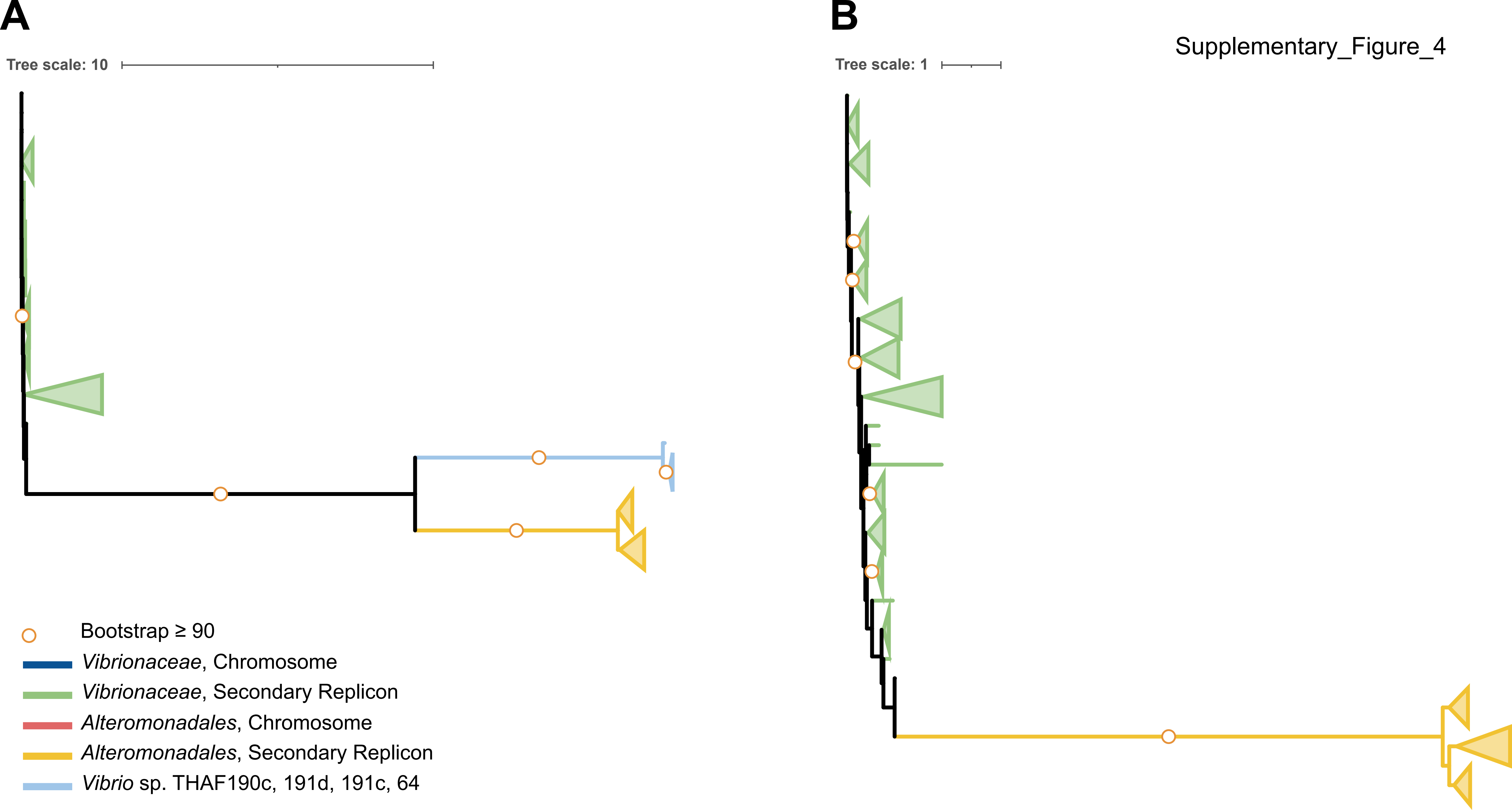

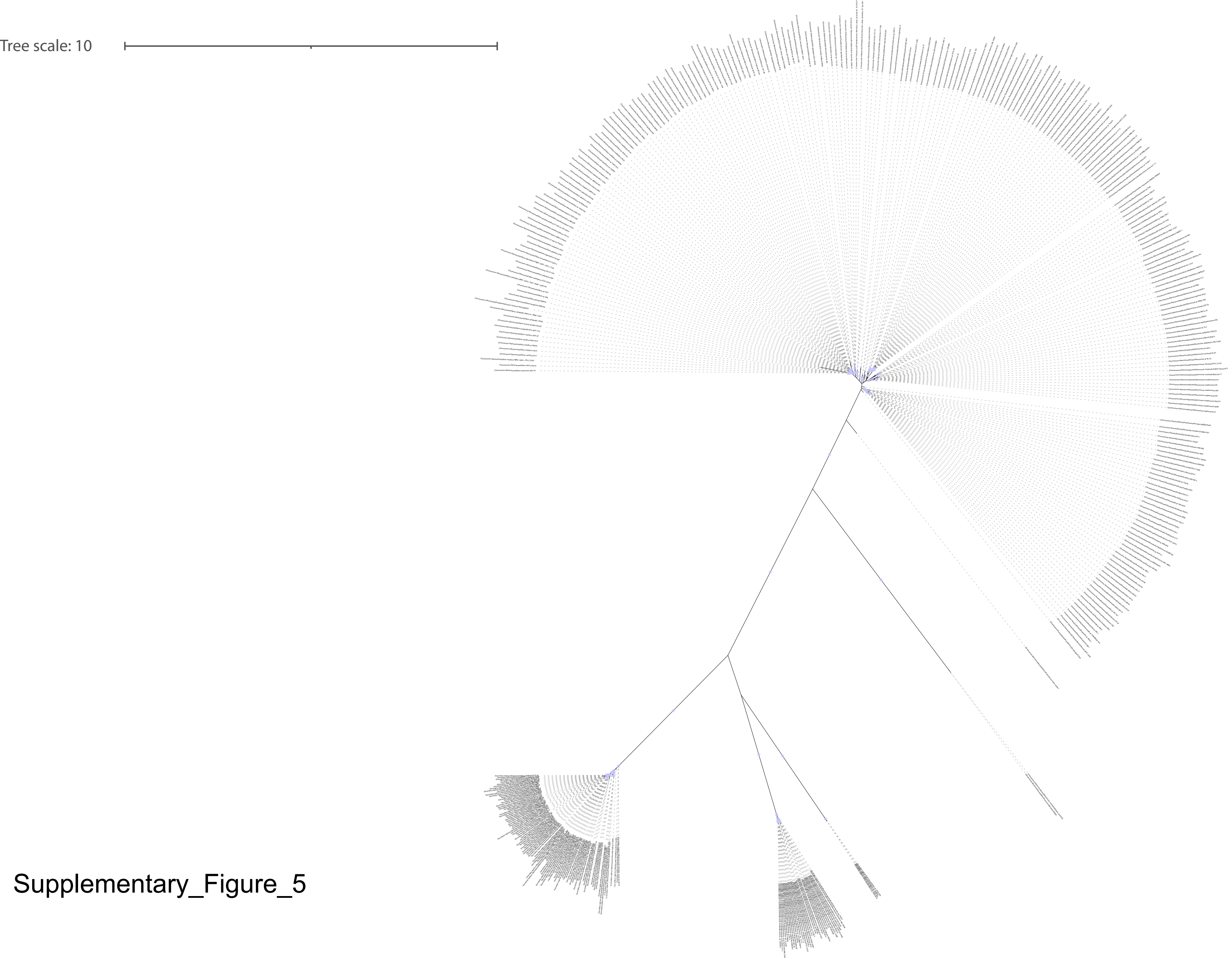

Supplementary_Figure_5

Tree scale: 10

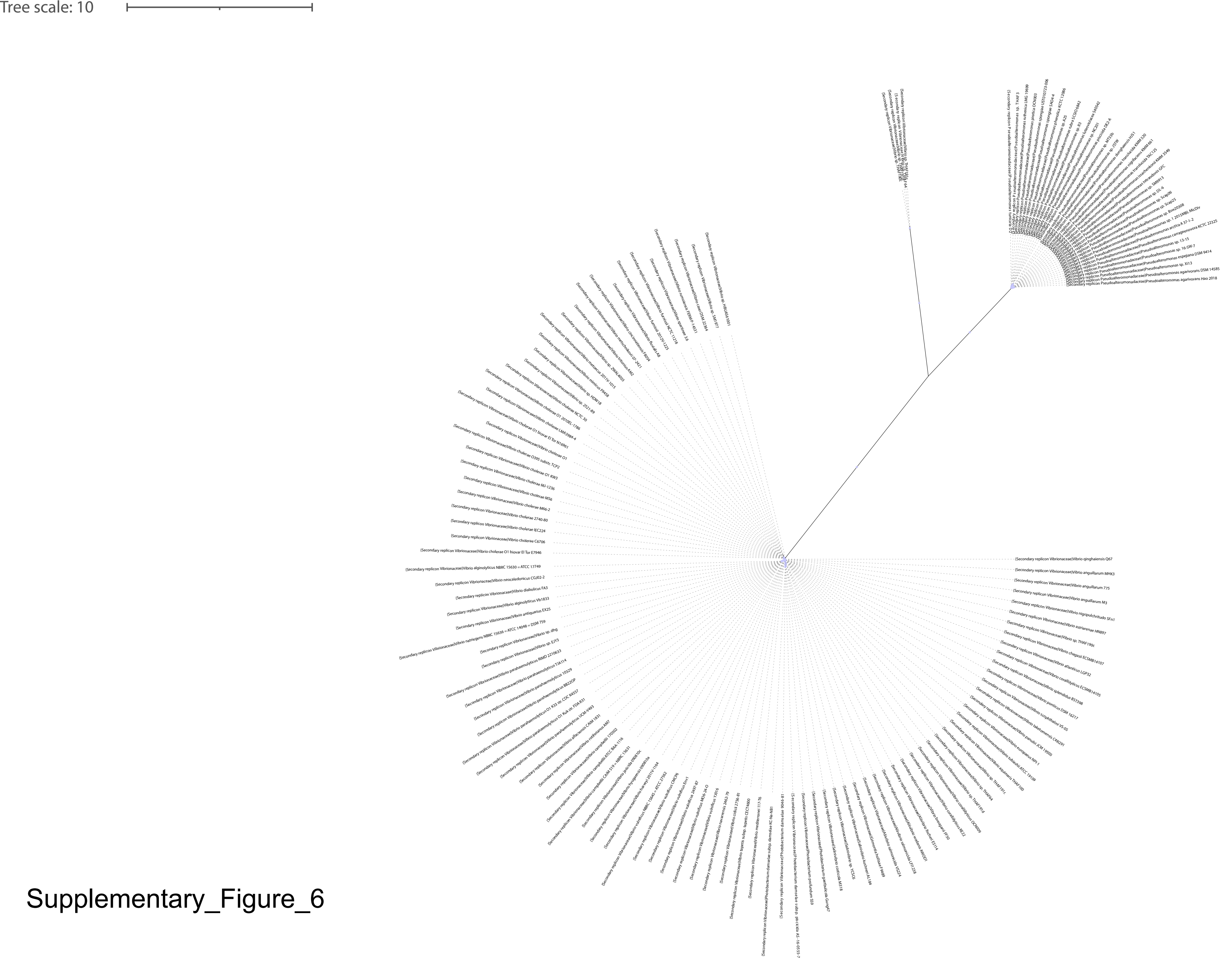

Supplementary_Figure_6

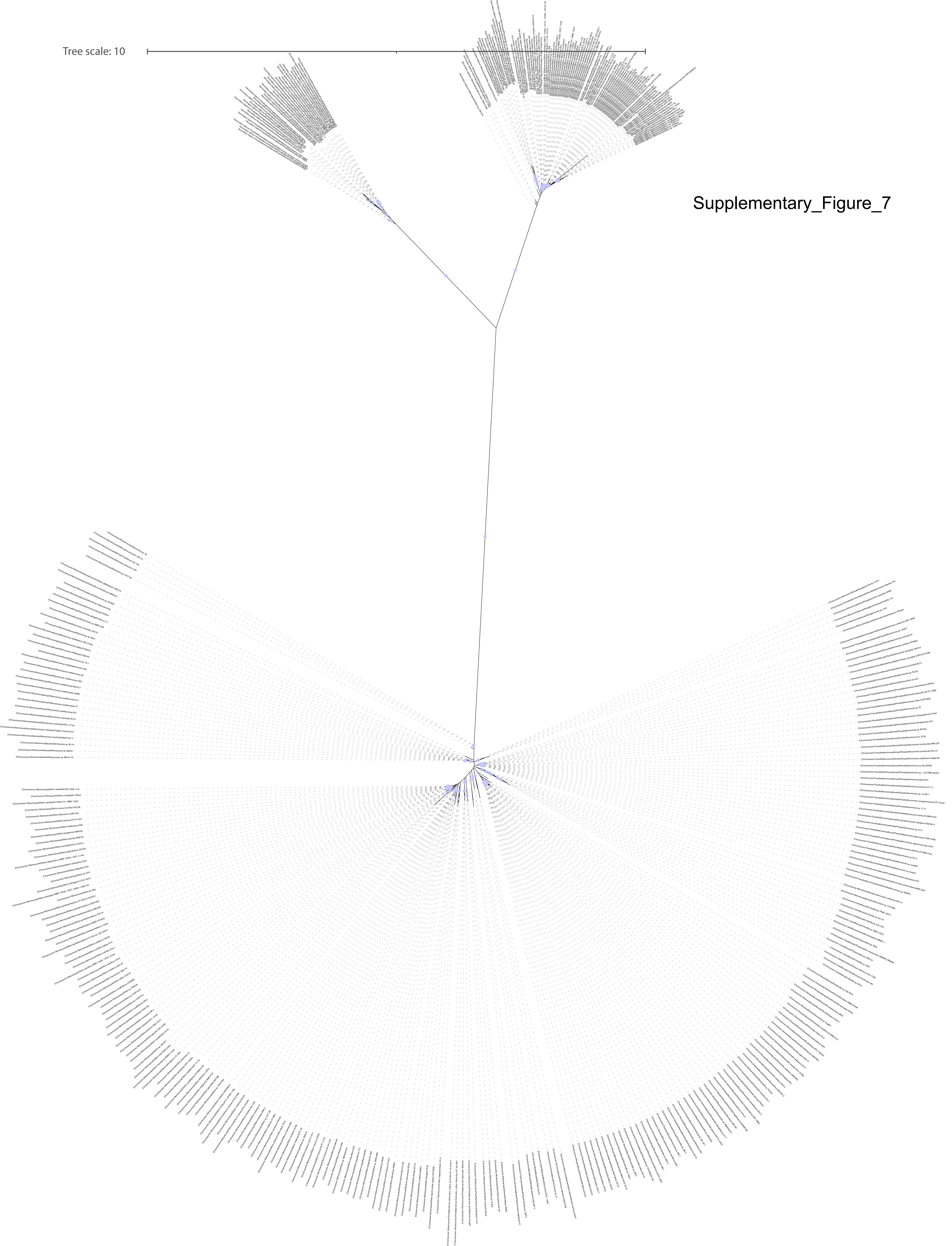

Supplementary_Figure_7

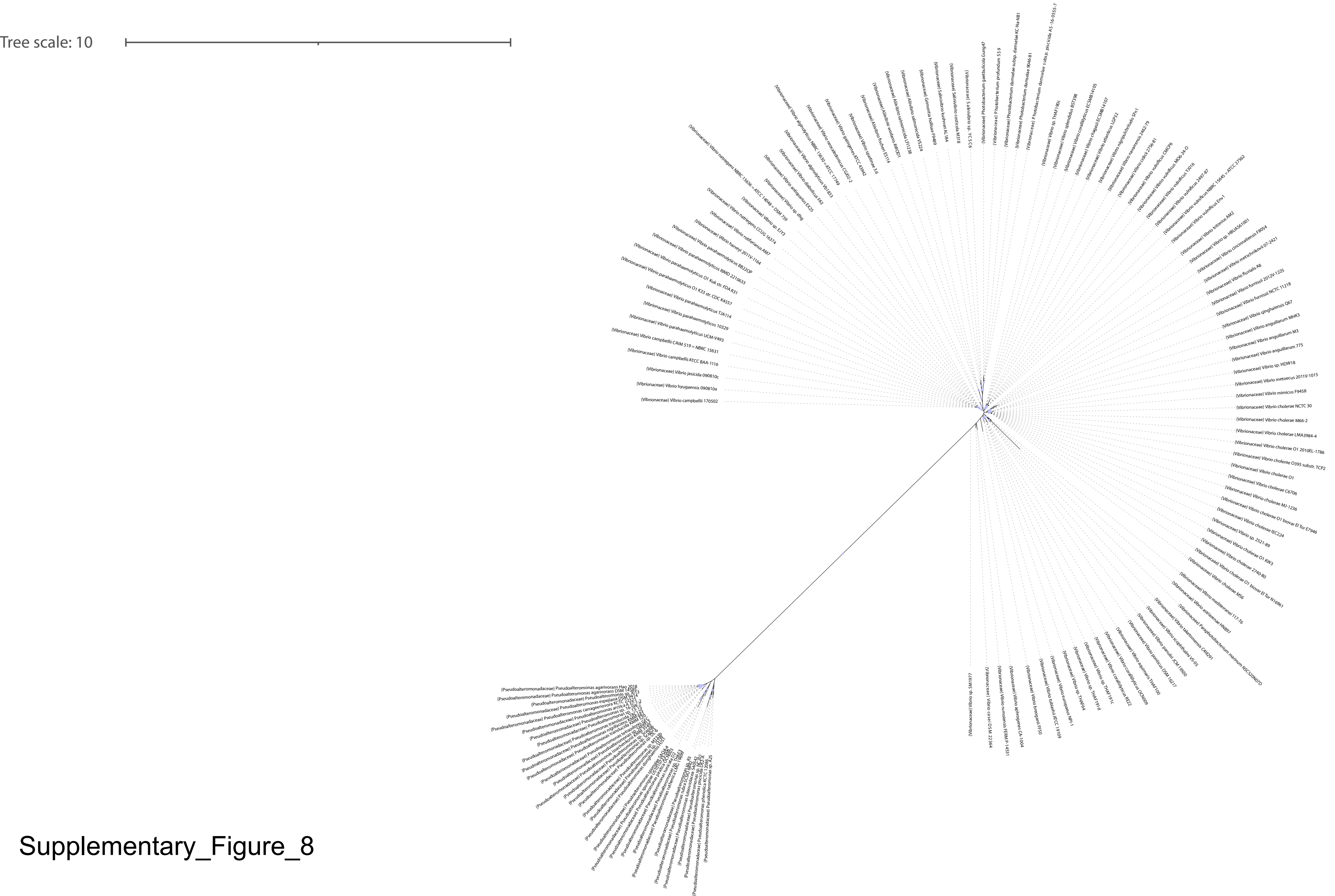

Supplementary_Figure_8

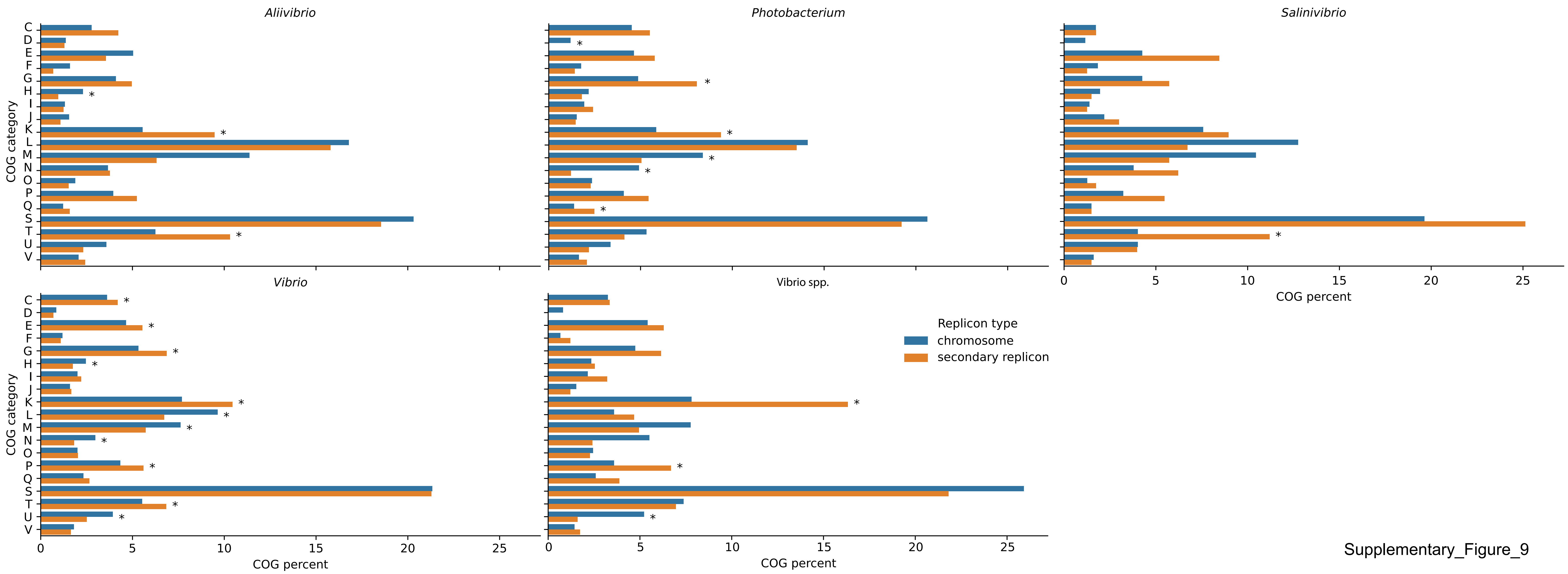

Supplementary_Figure_9

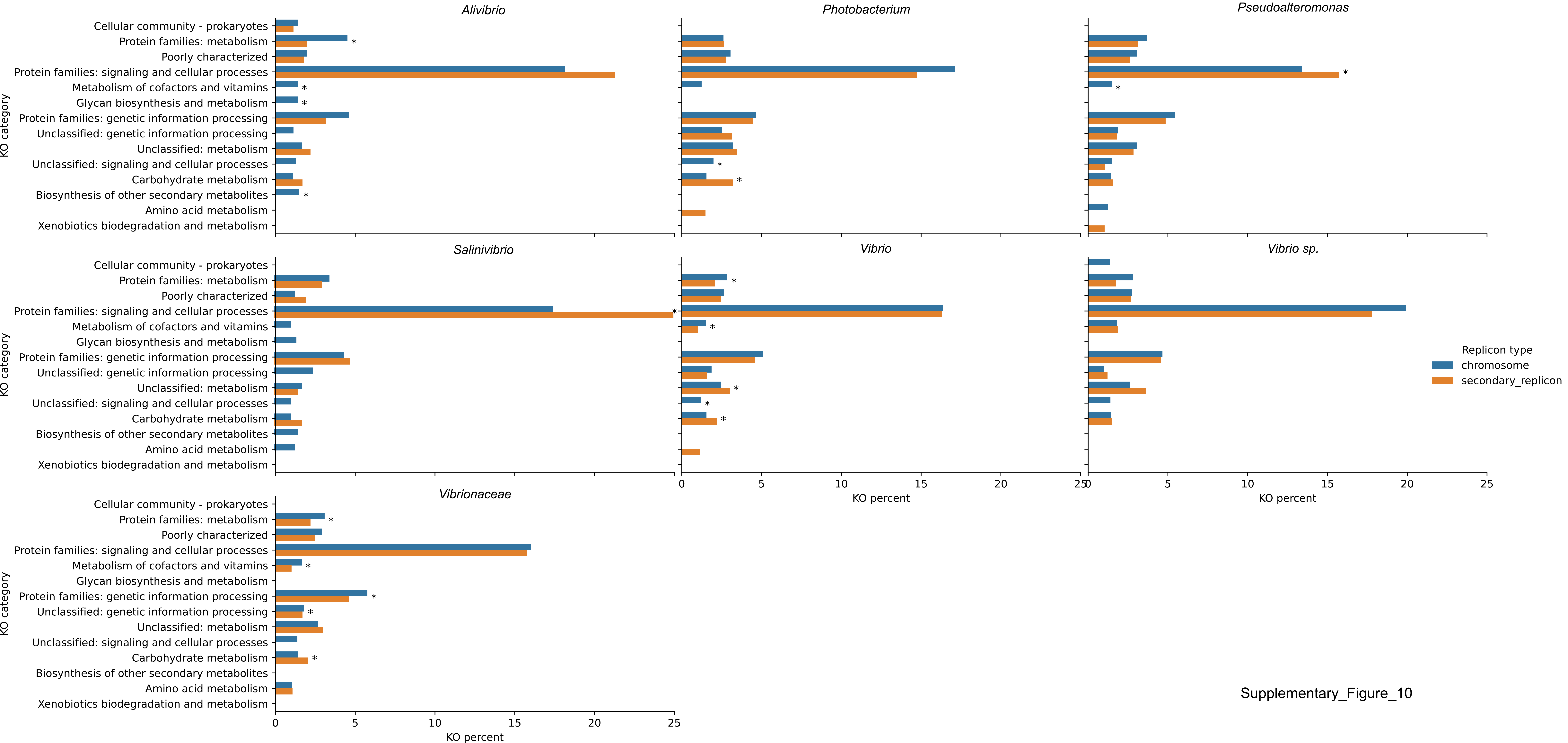

Supplementary_Figure_10
